# Impact of capacity building through learning, training, and coaching on agricultural innovation

**DOI:** 10.1371/journal.pone.0314004

**Published:** 2025-01-10

**Authors:** Learnmore Mwadzingeni, Martin Dandira, Dumisani Kutywayo, Liboster Mwadzingeni, Andrew Chiwawa, Mulala Danny Simatele

**Affiliations:** 1 Graduate School of Business, Bindura University of Science Education, Bindura, Zimbabwe; 2 Seed Co Limited, Research and Development, Harare, Zimbabwe; 3 Agricultural Research, Innovation, and Specialist Services (ARISS), Harare, Zimbabwe; 4 Department of Geography and Environmental Studies, University of the Witwatersrand, Johannesburg, South Africa; COMSATS University Islamabad - Wah Campus, PAKISTAN

## Abstract

Successful innovation requires employees to have intellectual and technical capacity. This study explored the effects of capacity building through educational learning, organizational training, and coaching on agricultural innovation. A sample of 142 operational-level agriculture scientists working within a public sector agricultural research organisation in Zimbabwe. Six key informants were also consulted. Descriptive statistics, factor analysis, principal component analysis (PCA), correlation analysis, and binary logistic regression analysis were employed. Results showed significant positive relationships between the variables stating that operational scientists receive adequate academic study opportunities from the research organisation and the one stating that they have relevant academic qualifications to help drive agriculture innovation (r = 0.421***). This reflects that once capacitated through educational learning, the employees gain the confidence and ability to innovate, which could be attributed to improved intelligence quotient (IQ). A significant positive relationship was also observed between the variables, stating that research employees required further training and that improving training would significantly improve innovation (r = 0.47***). Lastly, the variables, stating that direct supervisors offer adequate guidance to stimulate innovation positively correlated to the one stating that coaching has helped improve the operational staff’s innovativeness (P = 0.493***). This implies that efforts being put in by supervisors significantly contribute to innovation. Budgetary constraints were the leading challenge mentioned by 90.1% of the sample. Notably, there is a critical need to improve physical training workshops, exposure visits, and short courses to enhance innovativeness, as revealed by more than 70% of the respondents. The study also suggests cost-effective strategies to enhance capacity building and consequently stimulate innovation.

## 1. Background

Capacity building ensures innovation through collective actions which includes allowing researchers the flexibility to learn, train, and collaborate [1]. There is a need for an adaptive governance approach involving wide collaboration and capacity building among researchers and stakeholders within the agriculture and food systems value chain [2]. This requires knowledge about the factors that shape the direction and outcomes of transformation processes, including interactions across societal and sub-sector levels which are largely sustained through capacity building within learning organisations.

Learning organizations strive to ensure development, adaptation, and revolution in response to the ever-changing needs of clients and new challenges such as agricultural pests and diseases, decline in soil fertility, and recurrent droughts associated with climate change [3, 4]. This is achieved through learning; training and coaching, which prepares employees to be more productive, engaged, motivated and innovative [5–7]. About 50% of industrial and research employees require reskilling to be able to effectively use new technology since almost 33% of current skills and competencies are no longer considered crucial to today’s job requirements due to rapid technological changes [8]. In light of this, public and private sector research and regulatory organizations should ensure that all scientists and other technical staff are capacitated through academic advancements to learn new techniques in their respective disciplines. Learning can be accomplished at individual, group, and organizational levels [9–11].

Agricultural innovation entails the use of novel or existing products, processes, or ideas to increase efficiency, comparative advantage, sustainability, and adaptability of agriculture production, productivity, and profitability to enhance food and nutritional security [12]. It plays a pivotal role in the development of new demand-led technologies, products, and services, that enhances efficiency, reduce operating costs, and improve the profitability agricultural enterprises [13]. Similarly, the development of new markets requires innovative mind-sets and a culture that supports employees to see beyond the current status of the organization. This comes with the realization that the current agriculture business operating environment is rapidly developing due to rapidly evolving technologies, including robotics, automation, drones, and the “Internet of Things” that are being used to offer agricultural solutions.

Global crop yield losses associated with climate change-induced droughts, heat stress, frost, and crop pests, among other challenges, have been experienced by farmers over the past two decades [14–16]. If left unaddressed, this will cause up to 100% farm yield loss, resulting in food and nutritional insecurities [17]. Also, this makes the costs of production unbearable and increases environmental pollution through excessive use of agrochemicals, including insecticides and herbicides. To address these challenges, there is a need for the adoption of innovation which will increase the rate of release and commercialization of new technologies, including crop varieties, agro-chemicals, processes, and ideas to improve production and productivity. Such innovative solutions are promoted through capacity building, involving organisational learning, training, and coaching. This is supported by [18] who observed that incremental change in employee knowledge results in a change in their attitude and intentions to use environmentally sustainable work practices (ESWPs), which is an ingredient for sustainable agriculture innovation. However, according to the World Economic Forum, about 50% of industrial and research employees require reskilling to be able to embrace and use new technologies since almost 33% of current skills and competencies are no longer considered crucial to today’s job requirements due to rapid technological changes [8].

**Fig 1:**
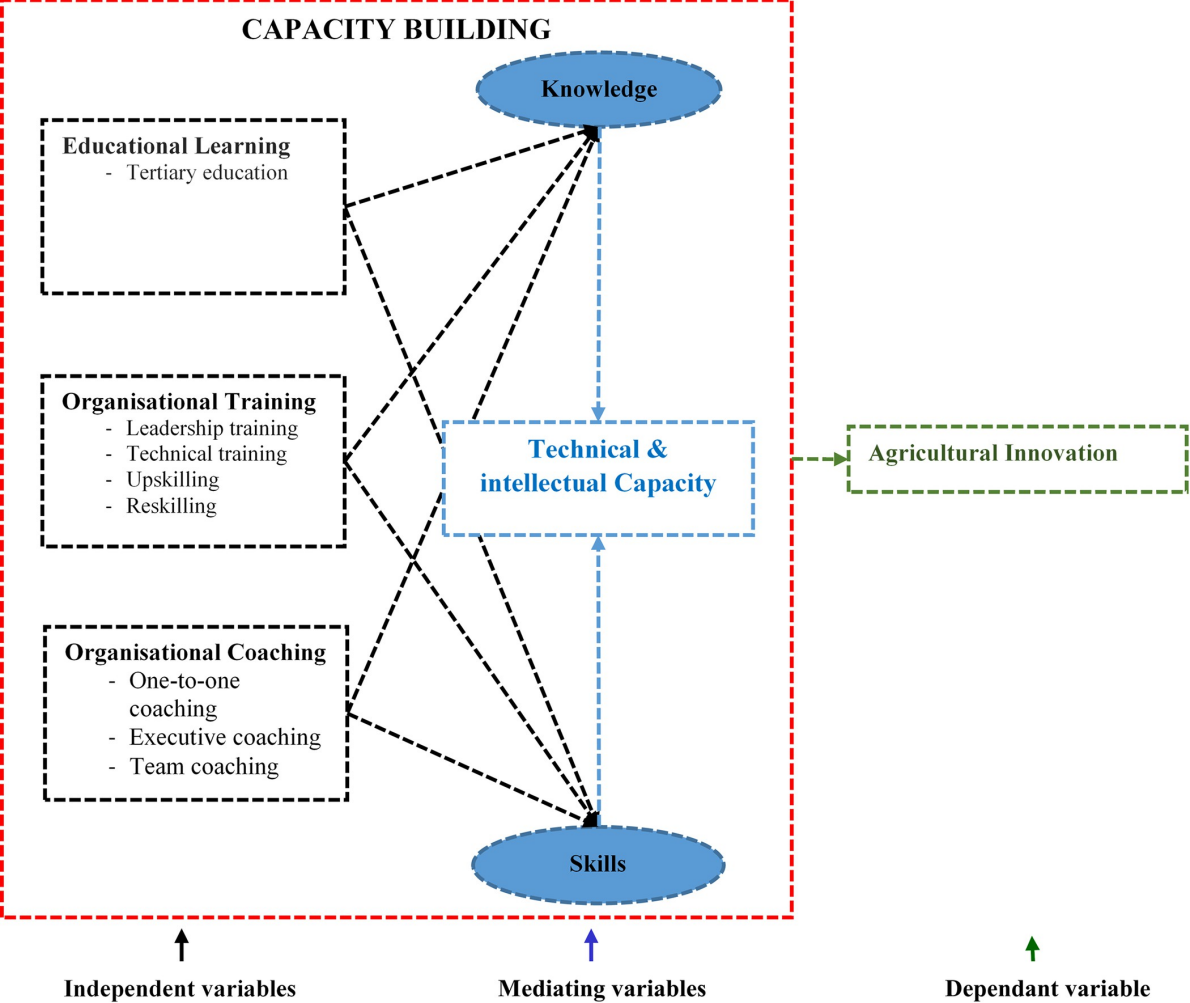
Conceptual framework of capacity building impact on innovation. **Independent variables**—Learning, training, and coaching can be manipulated by the organisation’s leadership to explore their respective impact on agricultural innovation. **Mediating variable**—Knowledge and skills are the direct outcomes of the independent variables that sum up to technical and intellectual capacity needed to achieve agricultural innovation. **Dependant variable**—Agricultural innovation is proposed to depend on the technical and intellectual capacity generated through learning, training, and coaching.

Innovation is an intrinsic aspect of agricultural transformation that is largely influenced by capacity building through organizational learning, training, and coaching. Public and private-sector researchers should therefore have the intellectual capacity to be able to play a pivotal role in pioneering agriculture innovations that counter new agricultural challenges. However, there is scanty scholarly literature and scientific evidence of the impact of capacity building through organisational learning, training, and coaching on agricultural innovation. This study therefore aimed to evaluate the effects of capacity building on agricultural innovation using a case study of the leading public sector agricultural research and regulatory organisation in Zimbabwe. The overall objective is to advocate for improved learning, training, and coaching to stimulate agriculture innovations aimed at improving food and nutritional security through improved production efficiency and productivity of food, feed, fibre, and fuel in the face of challenges associated with the current climate change. Fig 1 presents the conceptual framework proposed for this study. The framework suggests that educational learning, organisational training, and coaching generate the knowledge and skills needed to promote innovation. Learning, training, and coaching ultimately build employee knowledge and skills, which are the key mediating variables that should be applied to derive agricultural innovation.

## 2. Methods

This research targeted about 220 operational employees of the leading public sector agriculture research and regulatory department, the Agricultural Research, Innovation, and Specialist Services (ARISS), with 14 institutions situated in different locations in Zimbabwe. This was achieved through questionnaires distributed from the 17^th^ of April 2024 to the 14^th^ of May 2024. The directors and heads of institutes were considered key informants who responded to the key informant questionnaire with open-ended interview guides. The researcher carried out random sampling to reduce bias and save time since the entire population was too large to do the research on. A sample size of 142 employees from the research organisation’s stations across the country was used for statistical analysis. The main questionnaire was responded to by operational staff comprised of research officers including breeders, agronomists, economists, pathologists, entomologists, and biometricians, as well as their technicians. These employees were assumed to hold enough information about the research organisation’s agriculture innovation agenda and initiatives. The sample size was calculated using the following formula according to [19]:

Samplesize=[(Z−score)2xStdDevx(1−StdDev)]/(confidenceinterval)2

$$Sample\;size=\frac{\frac{Z^{2}*P(1-P)}{e^{2}}}{1+\frac{Z^{2}*P(1-P)}{e^{2}N}}$$

Where: Z-score = 1.96; Standard Deviation (*P*) = 0.5; confidence interval = 95%, Population size (N) = (220), Margin of error (*e*) = 5%. In addition to the sample used for statistical analysis, six key informants from the different institutes also provided their insights by completing the key informant questionnaire.

After the collection of all questionnaires, numerical and coded data was captured on a Microsoft Excel database. Raw data were edited through both field and in-house editing to detect and correct errors and omissions introduced through field or data entry. All data were analysed using descriptive statistics, factor analysis, Pearson’s correlation analysis, principle component analysis (PCA), and Binary Logistic Regression in the Statistical Package for the Social Sciences (SPSS) (https://www.ibm.com/products/spss-statistics). Factor analysis was used to condense and summarise the collected data into a distinct set of dimensional items that can influence the agricultural research institution’s decisions on capacity building to foster agricultural innovation. To test the adequacy of the sample size and the existence of relationships among variables, the Kaiser-Meyer-Olkin (KMO) and Bartlett’s tests were conducted. KMO values above 0.5 and significant Bartlett’s Tests below P = 0.05 for sample sizes between 100 and 200 indicate compact patterns of correlations that can be condensed into reliable and distinct factors and that the data is suitable for Factor Analysis [20–22]. This led to the use of Principal Component Analysis (PCA), Pearson’s product-moment correlation and Binary Logistic Regression Analysis. The logit model makes use of explanatory variables to estimate the probability that the response variable takes for each response variable [23, 24]. The binary logit model was used to identify the likelihood of factors to be associated with dichotomous dependent outcomes [25]. A binary regression model establishes the relationship between binary response variables and continuous and/or categorical explanatory variables [26]. The binary logit model was selected due to its ability to give an easy interpretation of variables [25]. In the model, a sample of *n* independent observations of (*y*_*i*_, *x*_*i*_), *i* = 1,2, …, *n*. Here, *y*_*i*_ represents a dichotomous response, and *x*_*i*_ represents the value of an independent variable of the *i*^*th*^ subject.

Therefore,

$$y_{i}=\left\{ \begin{array}{c} 1,\;yes\\ 0,\;No \end{array}\right.$$

The conditional mean *π*(*x*) for the expected value of *y* given *x* in binary regression is calculated using Eq [a]:

$$\pi (x)=\frac{e\beta_{0}+\beta_{1}x}{1+e\beta_{0}+\beta_{1}x}$$ [a]

Eq [a] was fit in the binary logistic regression model for dichotomous data ((0≤*π*(*x*)≤1 to estimate parameters *β*_0_ and *β*_1_. The natural log of the odds of subjects was predicted by the binary logistic regression model. The term of transformation of *π*(*x*) was calculated using Eq [b]:

$$logit\;\pi (x)=\ln[\frac{\pi (x)}{1-\pi (x)}]=\beta_{0}+\beta_{1}$$ [b]

When the predicted probability *π*(*x*) of code ‘1’ is higher and ‘0’ is lower, the logit value is continuous and ranges from −∞ to ∞. The conditional probability *P*(*Y* = 1|*x*) was provided for term *π*(*x*) was provided for Eq [a] and conditional probability *P*(*Y* = 0|*x*) was provided for term 1−*π*(*x*) for arbitrary parameters *β*_0_ and *β*_1_. For the pairs (*y*_*i*_, *x*_*i*_), the likelihood function is *π*(*x*_*i*_) if *y*_*i*_ = 1 and 1−*π*(*x*_*i*_) for *y*_1_ = 0; where the value of *π*(*x*) at *x*_*i*_ is *π*(*x*_*i*_) as illustrated in Eq [c]:

$$\pi {\left(x_{i}\right)}^{y_{i}}[-\pi (x_{i}){]}^{1-y_{i}}$$ [c]

The log function for logistic regression assuming independent observation was obtained using Eq [d]:

$$l(\beta )=\prod_{i=1}^{n}\pi (x_{i})[1-\pi (x_{i}){]}^{1-y_{i}}$$ [d]

Eq [d] can be represented as in Eq [e]

$$L(\beta )=\ln[l(\beta )]=\sum_{i=1}^{n}\{y_{i}\;\ln\left[\pi (x_{i}\right)]+(1+y_{i})\ln\left[1-\pi (x_{i})\right]\}$$ [e]

Two likelihood Eqs [f] and [g] were obtained concerning *β*_0_ and *β*_1_ following the differentiation of Eq [e] and solving for *β*.


$$\sum {[y}_{i}-\pi (x_{i})]=0$$ [f]

$$\sum {x_{i}[y}_{i}-\pi (x_{i})]=0$$ [g]

Eqs [f] and [g] are non-linear since its binary logistic regression, hence iterative method is needed for iterative weighted least square method. For this study, the binary logistic regression model is given in Eq [h]:

$$\ln\left[\frac{\pi {(x}_{i})}{1-\pi (x_{i})}\right]=\beta_{0}+\beta_{1}x_{i1}+\beta_{2}x_{i2}+\cdots +\beta_{n}x_{in}$$ [h]

Where: *x*_*i*1_, *x*_*i*2_,…,*x*_1*n*_–are categorical or continuous variables

The logistic regression equation for *π*(*x*_*i*_) in Eq [h] is

$$\pi (x_{i})=\frac{e^{\left(\beta_{0+}\beta_{1}x_{i1}+\beta_{2}x_{i2}+\cdots +\beta_{n}x_{in}\right)}}{1+e^{\left(\beta_{0}+\beta_{1}x_{i1}+\beta_{2}x_{i2}+\cdots +\beta_{n}x_{in}\right)}}$$

## 3. Results

### 3.1 Demographic variables of participants

High KMO above 0.5 and significant Bartlett’s Tests below P = 0.05 were obtained for all the datasets generated (Appendix 5 in S1 File). This indicated that the sample was adequate for factor analysis and was highly correlated according to [20, 22]. Appendix 6 1 summarises the demographic distribution of respondents of this study including the employee profession, age bracket, highest education level, management level, and years of employment in the organization, cross-tabulated against gender. Chi-Square Tests for the goodness of fit (X^2^) revealed statistically significant associations of gender with management level, and the number of years in the organization (P-value ≤ 0.1). A total of 142 respondents comprising 75(53.5%) males and 66(46.5%) females participated in this questionnaire survey, however, based on the chi-squared test, there was no significant difference in gender participation. Among the respondents, 55(40.4%) had at most a Diploma, 51 (37.5%) had Bachelor’s degrees, 29(21.3%) had Masters degrees and 1(0.7%) had PhDs. Also, there was a normal distribution of employees in different age groups with the majority being the middle-aged categories. Lastly, there was a balance of experienced and fairly new employees in the agriculture research organisation.

### 3.2 Case processing summary statistics for learning, training, and coaching variables

Table 1 provides the case processing summary statistics for the impact of educational learning, organisational training, and organisational coaching on innovation. Regarding educational learning, a greater proportion of the employees either agreed or strongly agreed that they received adequate academic study opportunities from the organization to promote innovation (59.8%) and that they had the relevant academic qualifications to help drive agriculture innovation (86.6%). However, most employees (91.5%) still require additional academic qualifications to improve their innovativeness. This is further confirmed by 92.3% of the employees who acknowledged that improving learning will significantly improve innovation. Regarding organizational training, a greater proportion of the employees either agreed or strongly agreed that training programs are effective in enhancing innovation (81%) and that the research organisation’s human resources (HR) department supports employees to attend relevant training opportunities (51.4%). Regarding organizational coaching, a greater proportion of the respondents at least agree that their direct supervisor offers them adequate guidance to stimulate innovation whenever needed (82.1%) and that coaching has helped improve their innovativeness (72.5%). However, about 60% of the employees revealed that they do not receive adequate coaching from external coaches from outside the organisation, with only 40.1% agreeing.

**Table 1:** Case processing summary statistics for the impact of educational learning, organisational training and organisational coaching on innovation within.

**1. Educational learning**	You receive adequate academic study opportunities from the organisation to promote innovation.	You have the relevant academic qualifications to help drive agriculture innovation.	You need additional academic qualifications to improve your innovativeness	Improving learning will significantly improve innovation.
Strongly Disagree	8	3	0	2
Disagree	18	2	1	1
Neutral	30	13	9	6
Agree	52	81	57	44
Strongly Agree	33	42	73	87
Missing value	1	1	2	2
**2. Organisational training**	Training programs are effective in enhancing innovation	The organisation’s HR supports you to attend relevant training opportunities.	You would require further training to be more innovative and productive	Improving training will significantly improve innovation.
Strongly Disagree	0	13	1	0
Disagree	6	18	0	2
Neutral	20	38	7	4
Agree	66	50	70	60
Strongly Agree	49	23	64	75
Missing	1	0	0	0
**3. Organisational coaching**	Your direct supervisor offers you adequate guidance to stimulate innovation whenever needed.	Coaching has helped improve your innovativeness.	You receive adequate coaching from external coaches from outside the organisation.	Improving coaching will significantly improve innovation.
Strongly Disagree	7	4	15	2
Disagree	7	6	25	3
Neutral	24	27	44	11
Agree	59	77	33	59
Strongly Agree	45	26	24	66
Missing value	0	2	1	1

### 3.3 PCA based on educational learning, training, and coaching variables

Appendix 7 in S1 File presents PCA showing the total variation explained based on responses on variables related to the effect of educational learning on innovation. Much of the variation observed was explained by the first Principal Component (PC 1), which accounted for 36.9% of the total variation. This was followed by PC 2, which accounted for 32.1%. Cumulatively the two PCs accounted for 69% of the total variation, which is quite substantial. Regarding organizational training, much of the variation observed was explained by PC 1 which accounted for 37% of the total variation. This was followed by PC 2, accounting for 27.37%. Cumulatively, the two PCs accounted for 64% of the total variation. PC 1 accounted for 44.5% of the total variation among organizational coaching variables, while PC 2 accounted for 24.4% of the total variation giving a cumulative figure of 68.9%.

Table 2 shows the rotated component matrix estimated loadings for variables related to the effect of educational learning, training, and coaching on innovation. The first PC 1 on learning variables is strongly correlated with the variables indicating that employees receive adequate academic study opportunities from the organisation to promote innovation and that employees have relevant academic qualifications to help drive agriculture innovation. The results show that agricultural researchers who receive adequate academic study opportunities are the ones who have the relevant academic qualifications to help drive agriculture innovation. The second PC is strongly correlated with two variables indicating that employees needed additional academic qualifications to improve their innovativeness and that improving learning will significantly improve innovation. Regarding organizational training, the first two variables on whether training programs are effective in enhancing innovation and if HR supports employees to attend relevant training opportunities had high positive loadings above 0.70 into PC 2. On the other hand, the last two variables on whether the employees require further training to be more innovative and productive and if improving training will significantly improve innovation had high positive loadings above 0.80 into PC 1.

**Table 2:** Rotated component matrix.

Educational learning variable	Component	
	1	2
a) You receive adequate academic study opportunities from the organisation to promote innovation.	**0.834**	-0.150
b) You have the relevant academic qualifications to help drive agriculture innovation.	**0.849**	0.094
c) You need additional academic qualifications to improve your innovativeness	-0.084	**0.805**
d) Improving learning will significantly improve innovation.	0.034	**0.810**
**Organisational training variables**	**1**	**2**
a) Training programs are effective in enhancing innovation	0.113	**0.725**
b) The organisation’s HR supports you to attend relevant training opportunities.	-0.090	**0.754**
c) You would require further training to be more innovative and productive.	0.855	**-0.032**
d) Improving training will significantly improve innovation.	0.852	**0.059**
**Organisational training variables**	**1**	**2**
a) Your direct supervisor offers you adequate guidance to stimulate innovation whenever needed.	0.702	-
b) Coaching has helped improve your innovativeness.	0.815	-
c) You receive adequate coaching from external coaches from outside the organisation.	0.587	-
d) Improving coaching will significantly improve innovation.	0.525	-

### 3.4 Correlation among variables

Some significant relationships were noted among variables related to the effect of educational learning, organisational training, and organisational coaching on innovation (Table 3). Notably, there was a significant positive relationship (r = 0.421***) between the variables stating that employees are receiving adequate academic study opportunities from the organisation and that they have relevant academic qualifications to help drive agriculture innovation. A significant negative relationship (r = -0.134*) was observed between employees receiving adequate academic study opportunities from the organisation and their need for additional academic qualifications to improve their innovativeness. Lastly, a significant positive relationship (r = 0.318***) was observed between employees needing additional academic qualifications to improve their innovativeness and their belief that improving learning will significantly improve innovation.

**Table 3:** Pearson’s correlation coefficients (r) describing associations among variables.

1. Educational learning variable	1a)	1b)	1c)	1d)
1a) You receive adequate academic study opportunities from the organisation to promote innovation.	1.000	0.421***	-0.134*	-0.065
1b) You have the relevant academic qualifications to help drive agriculture innovation.	0.421***	1.000	-0.004	0.032
1c) You need additional academic qualifications to improve your innovativeness	-0.134*	-0.004	1.000	0.318***
1d) Improving learning will significantly improve innovation.	-0.065	0.032	0.318***	1.000
**2. Organisational training variables**	**2a)**	**2b)**	**2c)**	**2d)**
2a) Training programs are effective in enhancing innovation	1.000	0.096	**0.036**	0.069
2b) The organisation’s HR supports you to attend relevant training opportunities.	0.096	1.000	**-0.033**	0.003
2c) You would require further training to be more innovative and productive.	0.036	-0.033	**1.000**	0.470***
2d) Improving training will significantly improve innovation.	0.069	0.003	0.470***	1.000
**3. Organisational training variables**	**3a)**	**3b)**	**3c)**	**3d)**
a) Your direct supervisor offers you adequate guidance to stimulate innovation whenever needed.	1.000	0.493***	**0.083**	0.183*
b) Coaching has helped improve your innovativeness.	0.493***	1.000	0.337***	0.173*
c) You receive adequate coaching from external coaches from outside the organisation.	0.083	0.337***	**1.000**	0.237**
d) Improving coaching will significantly improve innovation.	0.183*	0.173*	0.237**	1.000

Regarding organisational training, a significant positive relationship (r = 0.47***) between the employees requiring further training and their appreciation that improving training will significantly improve innovation was observed. Further, the variable suggesting that training programs are effective in enhancing innovation had a weak positive relationship with the variables stating that the organisation’s HR supports employees to attend relevant training opportunities (r = 0.096) and that employees would require further training to be more innovative and productive (r = 0.036), as well as the variable stating that improving training will significantly improve innovation (0.069).

Regarding coaching, a significant positive correlation (r = 0.493***) was observed between the variables, stating that direct supervisors offer adequate guidance to stimulate innovation whenever needed and that coaching has helped improve the employees’ innovativeness. This is further confirmed by the positive relationship (r = 0.337***) between the variables stating that employees receive adequate coaching from external coaches and that coaching has helped to improve employee innovativeness. The variable stating that improving coaching will significantly improve innovation is positively and significantly correlated to the other three variables stating that the employees’ direct supervisors offer guidance to stimulate innovation whenever needed (r = 0.183*), coaching has helped improve employee innovativeness (r = 0.173*), and that employees receive adequate coaching from external coaches from outside the organisation (r = 0.237**).

### 3.5 Strategies to enhance capacity building

**Fig 2:**
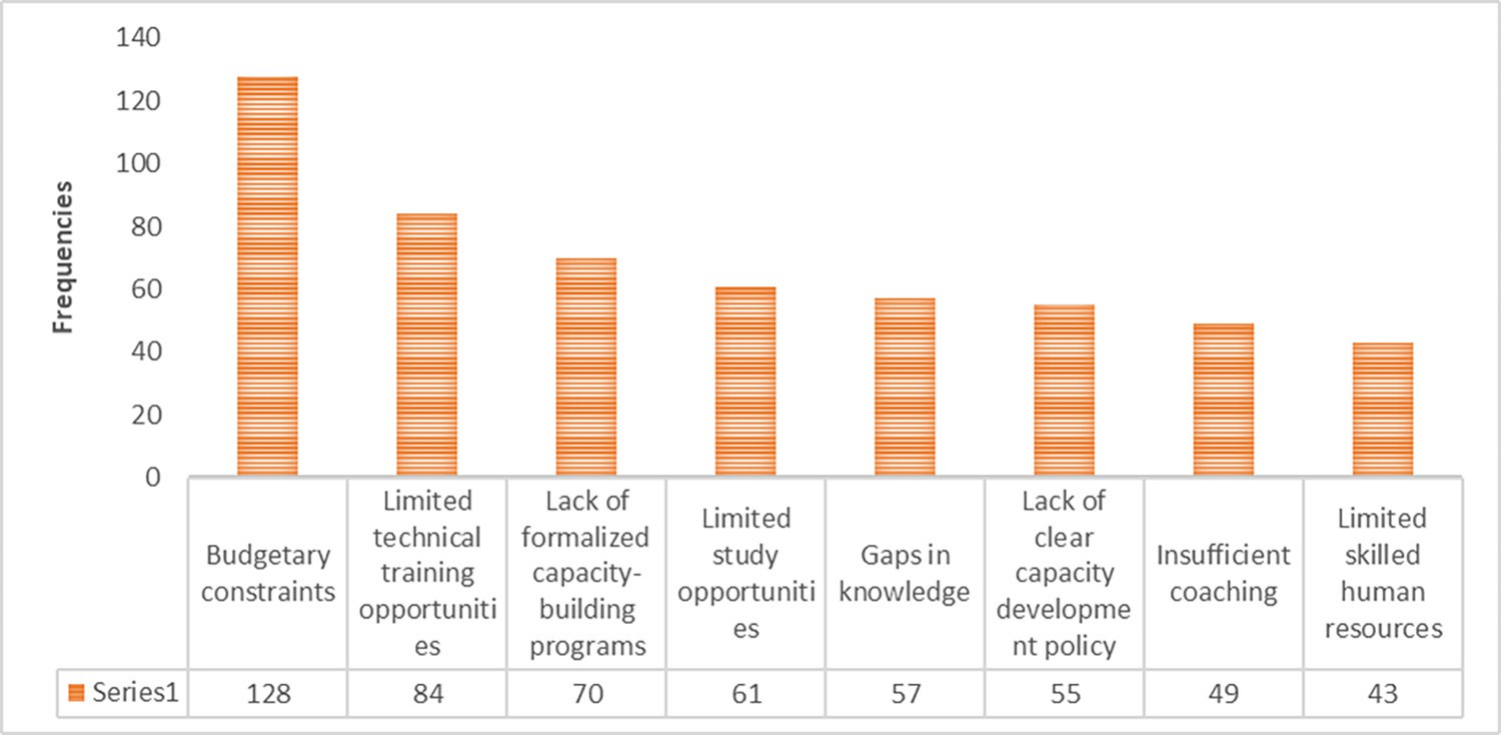
Challenges faced in delivering capacity building that may affect agriculture researchers’ innovation.

To be able to strategically prescribe strategies to enhance capacity building, the study explored the current challenges faced in delivering capacity building that may affect the agriculture innovation (Fig 2). Budgetary constraints were the leading challenge mentioned by 128 participants, constituting 90.10% of the sample. Limited technical training opportunities and a lack of formalized capacity-building programs followed on the list with 84 and 70 respondents, constituting 59.20% and 49.30% of the sample, respectively. Other challenges that were mentioned by 30% to 43% of the respondents were limited study opportunities, gaps in knowledge, a lack of clear capacity development policies, insufficient coaching, and limited skilled human resources.

## 3.6 Modes of capacity building suggested to be improved

**Fig 3:**
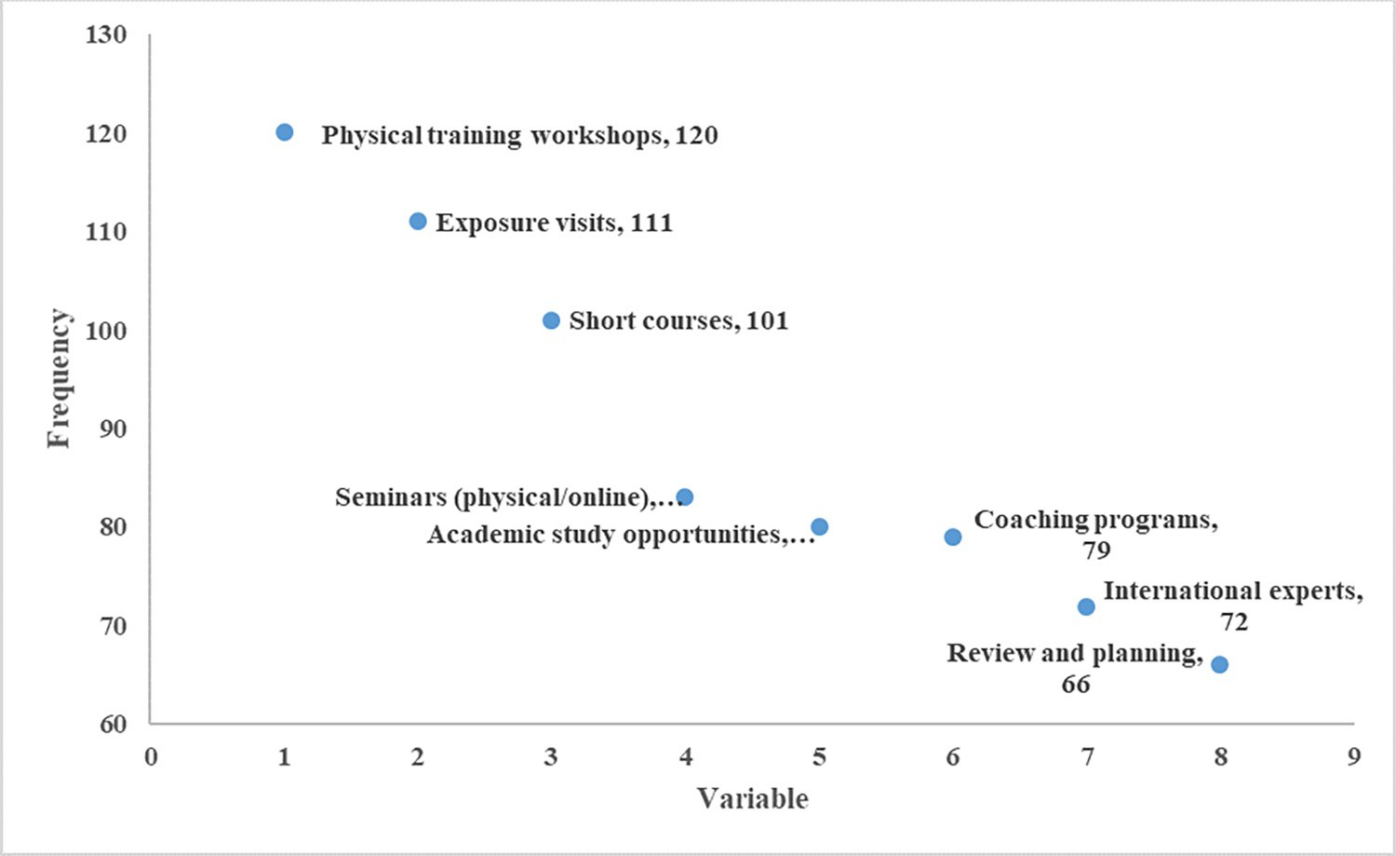
Modes of capacity building that need to be improved to promote agriculture innovation.

Fig 3 presents the modes of capacity building that need to be improved to promote agricultural innovation. There is a critical need to look into and improve the organization’s physical training workshops, exposure visits, and short courses as revealed by more than 70% of the respondents. This should be complemented by the enhancement of seminars (physical/online), providing academic study opportunities, enhancing coaching programs, and access to international experts as suggested by 50 to 60% of the respondents.

### 3.7 Strategies to improve the delivery of capacity building in agriculture research

**Fig 4:**
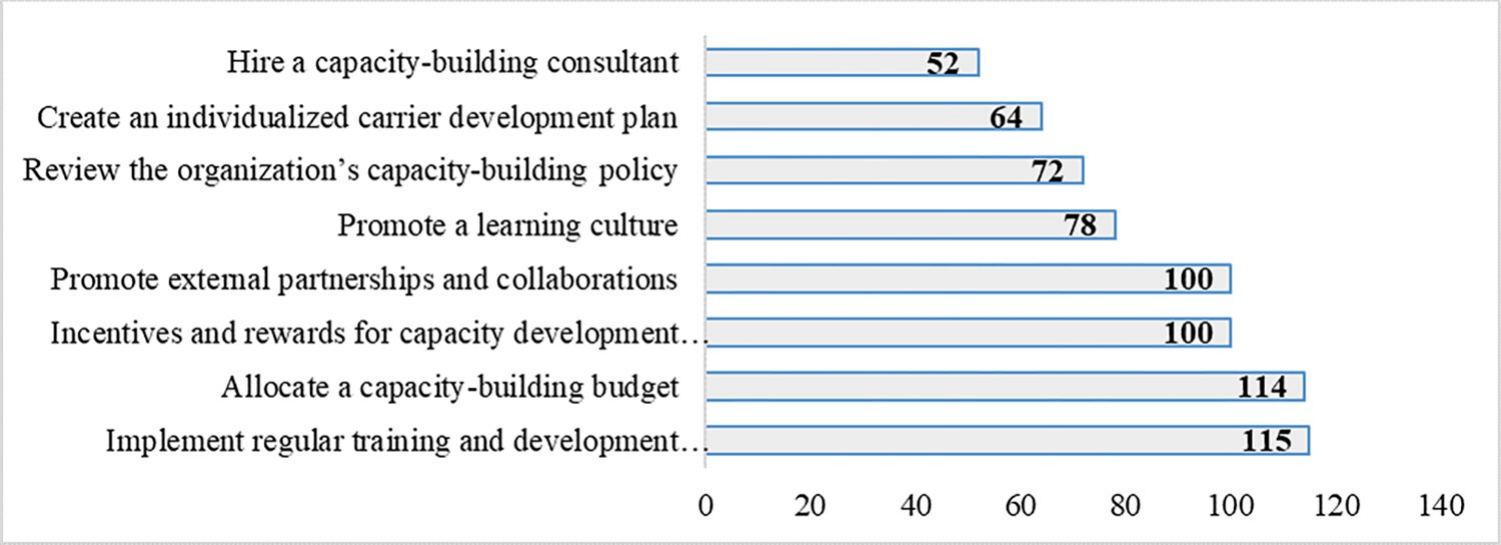
Strategies suggested to be implemented at the organisation to improve capacity building to boost agriculture innovation.

Fig 4 provides the strategies suggested to be implemented by the agriculture research organisation to improve capacity building. More than 70% of the respondents highlighted the need to implement regular training programs, allocate a capacity-building budget, offer incentives and rewards for capacity development achievements, and promote external collaborations. These need to be closely supported by promoting a learning culture and reviewing the organization’s capacity-building policy so that it talks to the organization’s strategy as reflected by about 50 to 55% of the respondents. Other strategies that need attention as indicated by 35% to 45% include creating an individualized career development plan and hiring capacity-building consultants.

### 3.8 Binary logistic regression of modes against strategies to improve capacity building

Binary logistic regression revealed negative Betas coefficients (B) for all significant relationships (Table 4). Academic study opportunities had a negative coefficient with hiring a capacity-building consultant, learning culture, and regular training. Seminars (physical or online) had a negative coefficient with reviewing capacity-building policies and promoting collaborations. Physical training workshops had a negative coefficient with the need to allocate a capacity-building budget and to offer incentives and rewards. Online training had a negative coefficient with review capacity-building policies and learning culture. International experts had a negative coefficient with reviewing capacity-building policies and regular training. Exposure visits had a negative coefficient with an individualized capacity-building plan, a review capacity-building policy, and regular training. Coaching programs had negative coefficients with reviewing capacity-building policies, incentives and rewards, promoting collaborations, and a learning culture. Short courses had negative correlations with allotting a capacity-building budget and an individualized capacity-building plan. Finally, review and planning had negative coefficients with hiring a capacity building consultant, allocating a capacity building budget, individualized capacity building plans, and regular training.

**Table 4:** Parameter estimates of the binary logistic regression model for the mode of capacity-building delivery to be improved (columns) against the strategies to enhance capacity-building delivery (rows).

	Academic study opportunities	Seminars (physical/ online)	Physical training workshops	On-line training	International experts	Exposure visits	Coaching programs	Short courses	Review & planning
Hire a CB consultant	-0.7*	-0.49	-0.47	-0.005	-0.40	-0.18	-0.98	0.412	-1.02*
Allocate a CB budget	0.47	-0.52	-0.97*	0.082	0.47	0.12	-0.18	-0.91*	-1.15*
Individualized CB plan	0.03	-0.212	-0.65	-0.243	-0.64	-1.36**	-0.27	-1.15**	-0.79*
Review CB policy	-0.64	-0.99**	-0.70	-1.30**	-1.29**	-0.97*	-1.66***	-0.50	0.05
Incentives and rewards	0.37	0.13	-1.23**	0.02	0.31	-0.66	-1.20**	-0.65	-0.39
Promote collaborations	-0.67	-1.00**	-0.52	-0.45	-0.869	-0.58	-1.09*	0.44	-0.84
Learning culture	-1.08**	-0.54	-0.75	-1.43**	-0.62	0.56	-1.17**	-1.23**	-0.42
Regular training	-1.04*	-0.43	0.44	0.12	-1.49**	-1.13**	0.30	-0.6	-1.79**
Constant	1.78***	2.02***	4.26***	0.99**	1.86***	3.36***	3.16***	2.77***	1.87***

CB = Capacity building

### 3.9 Suggestions to enhance capacity building from key informants

To enhance the delivery of capacity building through educational learning, the organisation should avail scholarships, funding opportunities, and grants for employees who develop innovative research projects to enrol in technical institutions of higher learning. There is a need for the research organization to ensure that the terms and conditions of MSc and PhD study leave promote continuous learning and that employees remain at motivating salaries as they will be advancing their studies for the benefit of the organization. Another way to promote capacity building through educational learning is to incentivize staff by regrading or promoting them as soon as they obtain higher qualifications, coupled with a significant improvement in remuneration.

Regarding training, there was an emphasis on the need to improve in-house training, workshops, and seminars to match up with new technology. This effort should be supported by an improvement in acquiring cutting-edge technologies that support the implementation of innovative ideas. Training and interaction/visits should be offered and supported equitably across the organisation’s employees and institutions since some stations and researchers felt left behind, particularly on foreign trips. Further, training should be tailor-made to address critical research areas of expertise, as some employees feel that training is mostly management-related without innovation-stimulating components.

Regarding organizational coaching, immediate supervisors require training to avoid just chasing results reports without offering the needed mentorship and guidance. Supervisors within the department also require adequate resources, including research facilities, for them to offer adequate guidance. To ensure that the whole process of capacity development works effectively, all research institutes need to be installed with functional internet facilities to ensure online learning, training, and research. Overall, there is a need to create a policy that allocates a budget and time for capacity-building programs tailored to the core business.

## 4. Discussions

The lack of significant difference in gender participation indicates that men and women had almost equal representation. This shows that the agriculture research organization complies with the gender policies of the country, the Southern African Development Community (SADC), the African Union, and the United Nations (UN) which advocate for a gender-just society that offers equal opportunities to men and women to achieve gender equality [27–30].

There was a healthy distribution of the age bracket of researchers, showing the organization’s commitment to return skills, and to nurture and groom new talent in agriculture research as was also recommended in the United States of America [31]. The normal distribution of employees in different age groups indicates a healthy distribution of employee age groups and experience since there is almost a balance between the young and older generations. A study on generational differences in the workforce carried out in the United States of America revealed that age significantly influences certain aspects of employee engagement [31]. A balance of age groups ultimately results in effective experience-sharing, coaching, and succession planning.

The downward trend of respondents observed as the level of education increases is normal since most technical staff are drawn from Diploma holders at entry level, followed by first degree then Masters who often occupy research officers’ positions, and lastly PhDs. There is a possibility that most PhD holders might have left the organization for greener pastures or may have been upgraded to executive positions. However, this is a reflection that there is a need for staff upgrades through academic advancement. Lastly, the balance of experienced and fairly new employees reveals a healthy trend for experience sharing.

He agriculture research organization is poised for transformative growth as reflected by the large proportion of employees who recognize that the training offered, and the support can improve innovation. However, there is a need for a critical assessment of the current training requirements by the employees because most of the employees (94.4%) at least agreed that they would require further training to be more innovative and productive. This was also confirmed by 95.1% of the employees acknowledging that improving training will significantly improve innovation as also highlighted by [32] who found that increased organizational training enhances both product and process innovation by 4.6% to 6.1% based on data on firms’ capacity development and innovation performance in Canada.

The concern of 60% of the employees who revealed that they do not receive adequate coaching from external coaches from outside the organisation needs to be addressed. If kept like this, the employees may find it difficult to catch up with the innovation pace of the private sector and other international public sector agriculture research institutions. Lack of external coaching could be the reason why most employees do not agree to the fact that improving coaching will significantly improve innovation, of which only less than 50% at least agreed to this variable. To change this perception, more external coaches should be brought in to guide employees to be innovative [33].

Results from PCA show that agricultural researchers who receive adequate academic study opportunities from the organisation are the ones who have the relevant academic qualifications to help drive agriculture innovation. These results supported the findings of a study carried out across 113 countries including Switzerland, Singapore, Sweden, and Finland [34]. This study postulates that there is a strong correlation between the creative learning ecosystem, education quality, and capacity to innovate. Therefore, organizations should prioritize investing in advancing agricultural training of those who are academically qualified taking advantage of their existing willingness to advance their academic qualifications. However, those who have not attained need to be incentivised to participate in the advancement of their academic qualification.

Based on the results agricultural researchers indicated that they need additional academic qualifications to improve their innovativeness and perceive that improving learning will significantly improve innovation. This shows that the agricultural researchers who prioritise academic qualifications believe that learning is a vehicle that drives innovation. This narrative shows that the drive that some employees have towards furthering their academic qualifications is based on their understanding of the importance of educational learning on innovation. Moreover, agriculture researchers believe that once they are capacitated, they will contribute effectively to the adoption of innovation, which supports the observations by [34]. Similarly, this team of agricultural researchers can easily support agricultural innovation based on their positive perception of capacity development.

According to the results, agricultural researchers who receive support from the organisation’s HR to attend relevant training opportunities are of the view that training programs are effective in enhancing innovation. Their drive to receive support from the organisation’s HR could be due to their compliance with the call for training because they appreciated its effectiveness. Further, employees’ perception that training will significantly improve innovation could have motivated them to advance their training. These results are supported by findings from a study on 10,366 firms in Eastern Europe and Central Asia that found that on-the-job training and education levels significantly and positively contribute to employees’ innovation capacity [35].

According to the results presented in Table 3, employees who are receiving adequate academic study opportunities are the ones who have relevant academic qualifications to help drive agriculture innovation. This reflects that once capacitated through learning, the employees gain the confidence and ability to innovate within their jobs. These findings are supported by a previous study which found that improved access to quality education promotes entrepreneurship and innovation as academic studies improve intelligence quotient (IQ) scores among other tools that derive creativity [36]. Further, those who have been capacitated have the confidence that they can innovate, hence, their need for additional qualifications gets reduced as they move towards reaching satisfaction. The relationships observed in this study show that employees who haven’t attained higher qualifications, strongly believe that if they get educated, their innovativeness will improve. Resultantly, there is enough room for the research organisation to boost its innovation agenda by capacitating employees through educational learning.

The findings that employees requiring further training appreciate that improving training will significantly improve innovation supports the findings by [32] who found that increased organizational training enhances product and process innovation by 4.6% to 6.1%. The agriculture research organisation should therefore invest in employee training to improve their innovativeness. Also, the relationships among variables reflected that employees appreciate that the efforts being put in by the organisation to provide the relevant training will drive innovation. Canadian organizations involved in research and development become more effective and innovative if they also invest in the training of their employees [37]. This also poses a challenge to the organisation as more employees seem to realize the importance of training on innovation through current programs, as reflected by the negative relationship (r = -0.03) between the training support offered by the organisation’s HR and the current need for further training to be more innovative.

The current results provide testimony that the effort being put in by supervisors and external coaches significantly contributes to employee innovation. External coaches often bring into the organization new ideas, skills, and technologies that improve the internal capacity to innovate. This is supported by a previous study which indicated that external coaches often come with vast experience from different organizations and will often be focused on coaching without any other duties, unlike internal coaches [33]. External coaching is an effective approach to managing resistance to change that is associated with innovation as revealed by [38]. Further, timely efforts to coach employees by direct supervisors or external coaches improve their capacity to innovate. This increase in innovation is attributed to psychological safety that is brought about through coaching [39]. The results are also supported by [40] who observed that coaching enhances employees’ innovativeness in Pakistan. This further confirms findings by [41] that strategic drivers such as user coordination and compatibility, which can be brought about through capacity building, are vital in manipulating the firms’ performance.

Since budgetary constraints were the leading challenge mentioned, the public-sector research institution needs to improve the budget allocated for capacity building and also find ways of improving funding. Further, there is a need for a re-look into the organization’s capacity-building policy and strategies to enhance the implementation of action items that ensure that employees receive the needed capacity-building. Reference can be made to the policy on capacity building adopted in Zimbabwe’s post-independence program where agriculture research officers were sent for MSc programs across the globe after probation to increase capacity. Executives and HRM of research organisations should look into ways of ensuring that these challenges are addressed effectively and uniformly across the institutions. To make capacity building through educational learning, training, and coaching complete, any grey areas regarding the current review and planning as well as online training should also be looked into as pointed out by less than half of the respondents. Planning and review meetings are part of the research planning and monitoring systems, which are critical in coaching agriculture research officers. The need to implement regular training and development programs was among the suggested strategies. Also, the research organisation needs to allocate a capacity-building budget and provide incentives and rewards for capacity development achievements. Lastly, there is a need to promote external partnerships and collaborations to capacitate employees and promote innovativeness. These suggestions support findings from previous studies previous studies that underscore the need to create a conducive environment for capacity building to enhance innovation [37, 42].

Binary logistic regression was used to help strategize cost-effective ways to execute capacity building based on the relationships among variables. Negative Betas coefficients (B) obtained from all significant relationships provide confidence that improving some of the predictor variables would result in a decrease in the need for some independent variables or strategies [23]. In this case, if the organisation is to increase the hiring of capacity-building consultants, that will significantly reduce the need for employees to access additional academic qualifications and will lower the need for review and planning. On the other hand, if a budget is allocated for capacity building it will most likely be channelled towards academic studies, hiring international experts, and exposure visits where positive betas coefficients are observed, consequently reducing the need for physical training workshops, short courses, and review and planning. If the organisation is to improve individualized capacity-building plans, the need for exposure visits, short courses, and review and planning will significantly be reduced. Improving incentives offered for capacity-building achievements will reduce the need for physical workshops and coaching programs as employees will be motivated to innovate through recognition. On the other hand, if the organisation promotes collaborations, that will supply employees with the necessary practical skills and knowledge. This will reduce the need for seminars and coaching programs. Similarly, adopting a learning culture will significantly reduce the appetite for academic study opportunities as more employees will get the much-needed education. This will also reduce the need for online training and short courses as recent advances in technology and new skills will be acquired through studies. Lastly, if the organisation is to adopt regular training, that will significantly reduce the need for academic study opportunities, international experts, and exposure visits since training will provide technical and soft skills that the other variables were supposed to take care of.

Research organisations should provide funding for scholarship programs. Scholarship programs provide access to a huge reservoir of knowledge and networks required for sustainable human resources development among organizations [43]. There is a need to offer incentives to promote capacity development. According to [44], intrinsic incentives motivate employees to be more innovative. Generally, effective incentives lead to reduced staff turnover, increased productivity, and high morale, as well as increased internal and external collaboration. This cultivates loyalty to the organization, teamwork, and a positive work environment, which leads to better organizational performance. Mostly, employees opt to leave the organisations after funding themselves, due to the slow pace of upgrades and remuneration increases or recognition after advancing their studies. Rewards and incentives will increase the zeal to learn and apply the knowledge gained innovatively.

The organization should support learning and training through the provision of functional Internet services. The request for functional internet facilities is supported by a study carried out in the United States of America, based on a county-level US broadband industry dataset, which found that access to internet facilities significantly increases innovation [45].

There is a need to enhance collaboration with the private sector, other government departments, universities, and international research organisations. Enhancing collaborations with the private sector, other government departments, universities, and other local or international research organizations would help to solve challenges revolving around the capacity-building budget and lack of funding. Results of a survey carried out in China based on 213 high-tech manufacturing enterprises confirmed that formal and informal external collaboration significantly contributes to improved innovation performance [46]. The results of this study provide valuable insights that can help to derive public-sector agricultural innovation. Further studies should include other value chain players such as agriculture processors, engineers, policymakers, and academia not included in the present study to provide a holistic view and to increase the target sample.

## 5. Conclusions and implications

The findings showed a significant positive relationship between capacity building through educational learning, organisational training, and organisational coaching with agricultural innovation. This reflects that effective generation of knowledge and skills builds technical and intellectual capacity to innovate. Research organisations should find ways to offer scholarship opportunities for their employees who develop innovative research projects and are eager to pursue their studies. There is a need to ensure that the terms and conditions of career advancement promote continuous learning and that employees remain motivated as they advance their studies. Human resources management should incentivize staff by upgrading them when they obtain higher qualifications to reduce staff turnover, increase productivity, and motivate innovation. In addition, training and coaching should be tailor-made to address critical research areas of expertise to ensure innovation. Moreover, immediate supervisors require training to avoid chasing results reports without offering adequate mentorship and guidance. Functional internet facilities and agricultural research stations ensures online learning, training, and research that derive innovation. Further, enhanced collaborations with the private sector and other local or international research organizations promotes public sector agricultural innovation and cost-sharing. Overall, policymakers should revisit capacity-building policy to ensure that learning, training, and coaching cope with global changes in climate and technologies impacting agriculture production and productivity.

## Supporting information

S1 File (DOCX)
