# Supplementary material for: Impact of capacity building through learning, training, and coaching on agricultural innovation
Source: PLoS One. 2025 Jan 10;20(1):e0314004. doi: 10.1371/journal.pone.0314004 (PMC11723611; doi:10.1371/journal.pone.0314004)
Supplement: S1 File — (DOCX) [file pone.0314004.s001.docx]

**Appendix 1: Main Questionnaire**

Questionnaire No:

**SECTION A: DEMOGRAPHIC CHARACTERISTICS OF RESPONDENTS**

1. May you please respond to the following questions by ticking the appropriate answer. Please make sure all the questions are answered.

| - 1. Institute |  | CBI |  | Seed Services | Other (Specify) ……………… | | | |
| --- | --- | --- | --- | --- | --- | --- | --- | --- |
|  |  |  |  |  |  | | | |
| - 1. Profession |  | Breeder |  | Agronomist | Other (specify) ……………… | | | |
|  |  |  |  |  |  |  |  |  |
| - 1. Gender |  | Male |  | Female |  |  |  |  |
|  |  |  |  |  |  |  |  |  |
| - 1. Age bracket |  | ≤ 30 years |  | 31-40 years |  | 41-50 years |  | ≥51 |
|  |  |  |  |  |  |  |  |  |
| - 1. Education level |  | ≤ Diploma |  | Bachelor’s |  | Masters |  | PhD |
|  |  |  |  |  |  |  |  |  |
| - 1. Management Level |  | Institution head |  | Operational Staff |  |  |  |  |
|  |  |  |  |  |  |  |  |  |
| - 1. Years in the organisation |  | <5 years |  | 5 to 10 years |  | 10 to 20 years |  | ≥21 |

Questionnaire No:

**SECTION B: IMPACT OF CAPACITY BUILDING ON INNOVATION**

Please indicate the extent to which you agree to the following statements.

**Key**: Strongly agree (5), Agree (4), Neutral (3), Disagree (2), Strongly disagree (1)

| - 1. Educational learning |  |  |  |  |  |
| --- | --- | --- | --- | --- | --- |
| **What is your personal view on the following statements?** | **Strongly**  **agree** | **Agree** | **Neutral** | **Disagree** | **Strongly disagree** |
| 1. You receive adequate academic study opportunities from the organisation to promote innovation. |  |  |  |  |  |
| 1. You have the relevant academic qualifications to help drive agriculture innovation. |  |  |  |  |  |
| 1. You need additional academic qualifications to improve your innovativeness |  |  |  |  |  |
| 1. Improving learning will significantly improve innovation. |  |  |  |  |  |
| Additional remarks: ……………………….. ………… …………… ……………… | | | | | |
|  |  |  |  |  |  |
| - 1. Organizational training |  |  |  |  |  |
| **What is your personal view on the following statements?** | **Strongly**  **agree** | **Agree** | **Neutral** | **Disagree** | **Strongly disagree** |
| 1. Training programs are effective in enhancing innovation |  |  |  |  |  |
| 1. The organisation’s HR supports you to attend relevant training opportunities. |  |  |  |  |  |
| 1. You would require further training to be more innovative and productive |  |  |  |  |  |
| 1. Improving training will significantly improve innovation. |  |  |  |  |  |
| Additional remarks: ……………………….. ………………………… ………… …… | | | | | |
|  |  |  |  |  |  |
| - 1. Organizational coaching |  |  |  |  |  |
| **What is your personal view on the following statements?** | **Strongly**  **agree** | **Agree** | **Neutral** | **Disagree** | **Strongly disagree** |
| 1. Your direct supervisor offers you adequate guidance to stimulate innovation whenever needed. |  |  |  |  |  |
| 1. Coaching has helped improve your innovativeness. |  |  |  |  |  |
| 1. You receive adequate coaching from external coaches from outside the organisation. |  |  |  |  |  |
| 1. Improving coaching will significantly improve innovation. |  |  |  |  |  |
| Additional remarks: ……………………….. ………………………… ………… …… | | | | | |

Questionnaire No:

**SECTION C: STRATEGIES TO ENHANCE CAPACITY BUILDING**

1. What are the challenges faced in delivering capacity building within your organisation that may affect the public sector’s innovation?

| **Challenges faced in delivering capacity building** | **Tick one or more options** |
| --- | --- |
| 1. Limited skilled human resources |  |
| 1. Gaps in knowledge |  |
| 1. Budgetary constraints |  |
| 1. Lack of clear capacity development policy |  |
| 1. Lack of formalized capacity-building programs |  |
| 1. Limited study opportunities |  |
| 1. Limited technical training opportunities |  |
| 1. Insufficient coaching |  |
| 1. Others (Specify)………………………………………….. …. |  |

1. Which modes of capacity building would you wish to be improved to promote public sector innovation within your organisation?

| **Capacity development mode** | **Tick one or more options** |
| --- | --- |
| 1. Academic study opportunities |  |
| 1. Seminars (physical/online) |  |
| 1. Physical training workshops |  |
| 1. On-line training |  |
| 1. International experts |  |
| 1. Exposure visits |  |
| 1. Coaching programs |  |
| 1. Short courses |  |
| 1. Review and planning |  |
| 1. Others (Specify) …………………………………………………. |  |

1. What strategies would you suggest to be implemented at your organisation to improve capacity building to boost public sector innovation?

| **Strategy to improve capacity building** | **Tick one or more options** |
| --- | --- |
| 1. Hire a capacity-building consultant |  |
| 1. Allocate a capacity-building budget |  |
| 1. Create an individualized carrier development plan |  |
| 1. Review the organization’s capacity-building policy |  |
| 1. Incentives and rewards for capacity development achievements |  |
| 1. Promote external partnerships and collaborations |  |
| 1. Promote a learning culture |  |
| 1. Implement regular training and development programs |  |
| 1. Others (Specify)………………………………………………… |  |

********THANK YOU*******

## Appendix 2: Key Informant Questionnaire

Questionnaire No:

1. May you please respond to the following questions by ticking the appropriate answer.

| - 1. Institute |  | CBI |  | Seed Services | Other (Specify) ……………… ….. |
| --- | --- | --- | --- | --- | --- |
|  |  |  |  |  |  |
| - 1. Director |  | Depute Director |  | Head of institution | Other (Specify) ……………… ….. |

1. Which modes of capacity building would you wish to be improved among your organisation’s employees to promote public sector innovation?

| **Capacity development mode** | **Tick one or more options** |
| --- | --- |
| 1. Academic study opportunities |  |
| 1. Seminars (physical/online) |  |
| 1. Physical training workshops |  |
| 1. International experts |  |
| 1. Exposure visits |  |
| 1. Coaching programs |  |
| 1. Short courses |  |
| 1. Review and planning |  |
| Others (Specify) ………………………… ……………… ………. …………. ……… | |

1. What are the limitations faced in delivering capacity building within your organisation and how can they be resolved?

…………………………………………………………………………………………………………………………………………………………………………………………………………………………………………………………………………………………………………………………………………………………………………………………………………

1. What strategies would you suggest to be implemented at your organisation to improve capacity building? ……………………………………………………………………………………………………………………………………………………………………………………………………………………………………………………………………………………………………………………………………………………………………………………………
2. If you were to influence policy on capacity building within the public sector, what would you include to enhance employee’s innovativeness?

……………………………………………………………………………………………………………………………………………………………………………………………… ……………………………………………………………………………………………

********THANK YOU********

## Appendix 3: Raw data on variables related to educational learning, organisational training and organisational coaching.

|  | Educational learning | | | | Organizational training | | | | Organizational coaching | | | |
| --- | --- | --- | --- | --- | --- | --- | --- | --- | --- | --- | --- | --- |
| ID | 2.1.a | 2.1.b | 2.1.c | 2.1.d | 2.2.a | 2.2.b | 2.2.c | 2.2.d | 2.3.a | 2.3.b | 2.3.c | 2.3.d |
| 3 | 5 | 5 | 5 | 5 | 5 | 5 | 5 | 5 | 5 | 5 | 5 | 5 |
| 4 | 4 | 4 | 5 | 5 | 5 | 5 | 5 | 5 | 5 | 5 | 4 | 5 |
| 6 | 4 | 4 | 5 | 4 | 2 | 4 | 4 | 5 | 3 | 2 | 2 | 4 |
| 7 | 5 | 4 | 4 | 5 | 4 | 5 | 4 | 4 | 4 | 4 | 4 | 3 |
| 10 | 4 | 4 | 4 | 4 | 4 | 4 | 4 | 5 | 4 | 4 | 3 | 3 |
| 11 | 4 | 4 | 5 | 4 | 4 | 5 | 4 | 4 | 5 | 5 | 3 | 4 |
| 12 | 4 | 4 | 5 | 4 | 5 | 5 | 4 | 4 | 4 | 5 | 4 | 5 |
| 14 | 1 | 3 | 3 | 5 | 4 | 4 | 4 | 4 | 4 | 4 | 4 | 5 |
| 18 | 4 | 4 | 4 | 5 | 5 | 4 | 4 | 5 | 4 | 5 | 4 | 5 |
| 19 | 2 | 5 | 5 | 5 | 2 | 2 | 5 | 5 | 2 | 3 | 4 | 5 |
| 20 | 5 | 5 | 4 | 5 | 5 | 4 | 5 | 5 | 5 | 5 | 5 | 5 |
| 22 | 3 | 4 | 5 | 5 | 4 | 4 | 5 | 4 | 4 | 4 | 4 | 4 |
| 24 | 4 | 4 | 5 | 5 | 4 | 3 | 4 | 4 | 4 | 3 | 2 | 5 |
| 25 | 5 | 5 | 4 | 5 | 3 | 4 | 5 | 5 | 5 | 4 | 3 | 5 |
| 26 | 5 | 4 | 5 | 3 | 4 | 3 | 3 | 4 | 5 | 3 | 4 | 4 |
| 27 | 4 | 4 | 3 | 4 | 4 | 4 | 4 | 4 | 4 | 4 | 3 | 4 |
| 28 | 5 | 5 | 4 | 4 | 5 | 4 | 5 | 5 | 5 | 5 | 5 | 5 |
| 29 | 5 | 5 | 4 | 5 | 4 | 4 | 5 | 5 | 5 | 5 | 3 | 4 |
| 31 | 4 | 4 | 5 | 5 | 4 | 4 | 5 | 5 | 4 | 4 | 4 | 5 |
| 32 | 3 | 5 | 5 | 5 | 5 | 1 | 4 | 5 | 3 | 3 | 3 | 3 |
| 33 | 4 | 4 | 4 | 5 | 4 | 3 | 5 | 5 | 4 | 4 | 3 | 4 |
| 34 | 4 | 5 | 5 | 4 | 3 | 3 | 4 | 4 | 4 | 4 | 2 | 4 |
| 36 | 4 | 4 | 4 | 4 | 3 | 4 | 5 | 4 | 4 | 4 | 2 | 4 |
| 37 | 3 | 5 | 5 | 5 | 4 | 3 | 4 | 4 | 4 | 4 | 3 | 4 |
| 38 | 3 | 4 | 5 | 5 | 5 | 3 | 5 | 5 | 4 | 4 | 3 | 5 |
| 39 | 4 | 5 | 4 | 5 | 3 | 3 | 4 | 5 | 4 | 4 | 3 | 5 |
| 40 | 3 | 3 | 5 | 5 | 5 | 3 | 5 | 5 | 4 | 3 | 1 | 5 |
| 41 | 2 | 4 | 4 | 4 | 5 | 1 | 4 | 4 | 5 | 4 | 1 | 4 |
| 42 | 2 | 4 | 4 | 4 | 5 | 2 | 4 | 4 | 5 | 4 | 1 | 4 |
| 43 | 2 | 4 | 4 | 4 | 5 | 1 | 4 | 4 | 5 | 4 | 1 | 4 |
| 44 | 2 | 4 | 4 | 4 | 5 | 1 | 4 | 4 | 5 | 4 | 1 | 4 |
| 46 | 2 | 3 | 3 | 3 | 5 | 1 | 4 | 4 | 5 | 4 | 1 | 3 |
| 50 | 4 | 4 | 4 | 4 | 4 | 4 | 4 | 4 | 4 | 4 | 4 | 4 |
| 51 | 3 | 4 | 5 | 5 | 4 | 3 | 4 | 5 | 3 | 3 | 4 | 5 |
| 52 | 4 | 4 | 4 | 4 | 4 | 2 | 4 | 4 | 3 | 2 | 2 | 2 |
| 53 | 5 | 5 | 5 | 5 | 5 | 4 | 5 | 4 | 5 | 5 | 5 | 5 |
| 54 | 3 | 5 | 4 | 5 | 3 | 4 | 4 | 5 | 4 | 4 | 3 | 5 |
| 55 | 5 | 3 | 4 | 4 | 4 | 5 | 5 | 4 | 4 | 3 | 3 | 5 |
| 56 | 3 | 4 | 4 | 5 | 3 | 3 | 4 | 4 | 4 | 4 | 5 | 4 |
| 57 | 4 | 4 | 4 | 3 | 4 | 4 | 4 | 4 | 3 | 4 | 4 | 4 |
| 58 | 4 | 4 | 4 | 4 | 5 | 2 | 5 | 5 | 4 | 4 | 2 | 4 |
| 59 | 4 | 5 | 5 | 4 | 5 | 5 | 4 | 4 | 4 | 4 | 3 | 3 |
| 60 | 5 | 3 | 5 | 5 | 5 | 5 | 3 | 5 | 5 | 5 | 2 | 5 |
| 61 | 5 | 4 | 5 | 4 | 4 | 5 | 5 | 4 | 5 | 4 | 2 | 4 |
| 62 | 3 | 4 | 5 | 5 | 4 | 4 | 5 | 5 | 5 | 3 | 2 | 4 |
| 63 | 3 | 4 | 5 | 5 | 4 | 4 | 5 | 5 | 4 | 3 | 2 | 4 |
| 65 | 4 | 4 | 5 | 4 | 3 | 3 | 4 | 4 | 3 | 3 | 4 | 4 |
| 66 | 5 | 5 | 4 | 5 | 3 | 4 | 5 | 5 | 5 | 4 | 3 | 5 |
| 67 | 5 | 4 | 4 | 5 | 5 | 4 | 4 | 4 | 5 | 4 | 1 | 4 |
| 68 | 3 | 5 | 5 | 5 | 5 | 2 | 5 | 5 | 5 | 5 | 3 | 5 |
| 69 | 4 | 5 | 5 | 5 | 4 | 1 | 4 | 4 | 3 | 4 | 2 | 4 |
| 70 | 4 | 3 | 5 | 5 | 3 | 2 | 3 | 3 | 2 | 3 | 3 | 3 |
| 71 | 4 | 5 | 5 | 5 | 4 | 4 | 4 | 4 | 4 | 3 | 3 | 4 |
| 72 | 4 | 4 | 5 | 5 | 5 | 1 | 5 | 5 | 1 | 4 | 5 | 5 |
| 73 | 3 | 3 | 4 | 4 | 5 | 3 | 4 | 5 | 5 | 4 | 3 | 4 |
| 74 | 4 | 4 | 5 | 5 | 5 | 2 | 5 | 4 | 3 | 3 | 5 | 5 |
| 75 | 4 | 3 | 5 | 5 | 5 | 3 | 5 | 5 | 4 | 4 | 3 | 4 |
| 77 | 5 | 4 | 4 | 4 | 5 | 4 | 5 | 5 | 4 | 5 | 5 | 4 |
| 80 | 4 | 4 | 5 | 4 | 4 | 5 | 3 | 3 | 3 | 4 | 3 | 3 |
| 81 |  | 4 | 3 | 4 | 5 | 5 | 3 | 5 | 3 | 4 | 2 | 4 |
| 82 | 4 | 5 | 4 | 5 | 4 | 4 | 4 | 4 | 5 | 5 | 2 | 5 |
| 84 | 3 | 3 | 4 | 4 | 5 | 1 | 5 | 5 | 1 | 4 | 5 | 5 |
| 85 | 4 | 4 | 4 | 5 | 5 | 3 | 4 | 4 | 4 | 4 | 3 | 3 |
| 86 | 4 | 4 | 4 | 4 | 5 | 3 | 5 | 4 | 4 | 4 | 3 | 4 |
| 88 | 5 | 5 | 5 | 5 | 4 | 5 | 5 | 5 | 5 | 5 | 5 | 5 |
| 89 | 2 | 4 | 5 | 5 | 2 | 3 | 5 | 5 | 5 | 4 | 2 | 5 |
| 91 | 4 | 5 | 5 | 2 | 4 | 2 | 5 | 4 | 4 | 5 | 4 | 1 |
| 93 | 2 | 3 | 5 | 5 | 4 | 3 | 4 | 5 | 4 | 4 | 3 | 4 |
| 94 | 3 | 4 | 5 | 5 | 4 | 5 | 5 | 5 | 5 | 4 | 4 | 5 |
| 95 | 2 |  |  |  | 5 | 5 | 4 | 4 | 5 | 4 | 5 |  |
| 96 | 3 | 4 | 5 | 5 | 5 | 3 | 4 | 4 | 3 | 3 | 4 | 4 |
| 98 | 3 | 4 | 5 | 5 | 4 | 4 | 5 | 5 | 5 | 4 | 5 | 4 |
| 103 | 4 | 4 | 4 | 4 | 5 | 5 | 5 | 5 | 2 | 4 | 1 | 5 |
| 104 | 4 | 4 | 4 | 1 | 4 | 4 | 1 | 4 | 4 | 3 | 3 | 4 |
| 106 | 4 | 5 | 4 | 4 | 4 | 3 | 5 | 5 | 5 | 4 | 3 | 5 |
| 107 | 5 | 5 | 4 | 5 | 5 | 4 | 4 | 5 | 4 | 5 | 5 | 5 |
| 108 | 1 | 1 | 4 | 4 | 4 | 2 | 4 | 5 | 2 | 4 | 5 | 5 |
| 109 | 5 | 4 | 4 | 4 | 4 | 4 | 4 | 4 | 5 | 4 | 4 | 4 |
| 109 | 1 | 1 | 4 | 5 | 4 | 1 | 5 | 5 | 1 | 5 | 1 | 1 |
| 110 | 1 | 1 | 5 | 5 | 5 | 5 | 5 | 5 | 1 | 1 | 1 | 5 |
| 111 | 1 | 4 | 5 | 5 | 4 | 4 | 4 | 4 | 2 | 2 | 2 | 2 |
| 112 | 4 | 4 | 5 | 5 | 4 | 4 | 5 | 5 | 4 | 4 | 5 | 5 |
| 114 | 4 | 5 | 2 | 2 | 4 | 4 | 4 | 4 | 5 | 4 | 2 | 4 |
| 116 | 1 | 5 | 5 | 5 | 4 | 3 | 5 | 5 | 1 | 4 | 3 | 5 |
| 117 | 2 | 3 | 4 | 5 | 5 | 1 | 5 | 5 | 3 | 3 | 3 | 5 |
| 118 | 4 | 5 | 3 | 5 | 5 | 3 | 4 | 4 | 3 | 4 | 5 | 5 |
| 119 | 4 | 4 | 5 | 5 | 4 | 4 | 4 | 4 | 4 | 4 | 4 | 4 |
| 120 | 5 | 5 | 4 | 5 | 5 | 4 | 4 | 4 | 3 | 4 | 5 | 5 |
| 121 | 4 | 4 | 5 | 5 | 4 | 4 | 5 | 5 | 4 | 5 | 5 | 5 |
| 122 | 5 | 4 | 5 | 5 | 4 | 4 | 4 | 4 | 3 | 3 | 4 | 5 |
| 123 | 3 | 4 | 5 | 5 | 5 |  | 5 | 5 | 3 | 4 | 3 | 4 |
| 123 | 5 | 5 | 5 | 5 | 5 | 2 | 5 | 5 | 5 | 4 | 5 | 5 |
| 124 | 5 | 5 | 4 | 5 | 5 | 4 | 5 | 4 | 5 | 5 | 4 | 4 |
| 125 | 1 | 4 | 5 | 5 | 2 | 4 | 5 | 5 | 4 | 1 | 1 | 5 |
| 126 | 3 | 4 | 5 | 5 | 5 | 3 | 5 | 5 | 4 | 4 | 2 | 5 |
| 127 | 5 | 5 | 5 | 5 | 3 | 1 | 5 | 5 | 4 | 4 | 4 | 5 |
| 128 | 2 | 4 | 4 | 4 | 4 | 3 | 4 | 4 | 1 | 1 | 2 | 4 |
| 129 | 2 | 4 | 5 | 5 | 2 | 2 | 5 | 5 | 3 | 3 | 1 | 4 |
| 130 | 3 | 4 | 5 | 5 | 4 | 5 | 5 | 4 | 5 | 4 | 3 | 5 |
| 131 | 4 | 4 | 5 | 5 | 3 | 3 | 5 | 5 | 4 | 4 | 3 | 5 |
| 132 | 5 | 4 | 4 | 4 | 4 | 4 | 4 | 4 | 5 | 4 | 4 | 4 |
| 152 | 3 | 4 | 5 | 4 | 4 | 3 | 4 | 4 | 4 | 3 | 4 | 4 |
| 153 | 3 | 3 | 4 | 5 | 3 | 3 | 4 | 4 | 3 | 4 | 3 | 5 |
| 154 | 2 | 4 | 5 | 5 | 3 | 3 | 5 | 5 | 3 | 3 | 2 | 5 |
| 155 | 2 | 4 | 4 | 5 | 4 | 4 | 5 | 5 | 4 | 4 | 1 | 5 |
| 156 | 3 | 4 |  |  |  | 3 | 5 |  | 5 |  |  | 5 |
| 157 | 3 | 4 | 5 | 5 | 4 | 3 | 4 | 5 | 4 | 4 | 3 | 4 |
| 158 | 4 | 4 | 5 | 5 | 4 | 3 | 5 | 5 | 3 | 4 | 2 | 5 |
| 159 | 2 | 4 | 5 | 5 | 3 | 2 | 5 | 5 | 3 | 3 | 2 | 5 |
| 161 | 5 | 4 | 5 | 5 | 4 | 3 | 5 | 5 | 4 | 4 | 4 | 4 |
| 162 | 3 | 4 | 4 | 4 | 3 | 2 | 4 | 4 | 3 | 2 | 2 | 4 |
| 163 | 2 | 2 | 5 | 5 | 3 | 2 | 4 | 5 | 2 | 2 | 2 | 4 |
| 164 | 4 | 5 | 4 | 5 | 5 | 5 | 4 | 4 | 5 | 4 | 5 | 4 |
| 166 | 4 | 5 | 5 | 5 | 5 | 4 | 5 | 5 | 4 | 5 | 3 | 5 |
| 167 | 1 | 4 | 5 | 3 | 5 | 2 | 5 | 5 | 5 | 3 | 1 | 4 |
| 168 | 3 | 4 | 5 | 4 | 4 | 4 | 4 | 4 | 4 | 4 | 4 | 4 |
| 169 | 4 | 4 | 2 | 4 | 4 | 2 | 4 | 2 | 2 | 4 | 2 | 2 |
| 170 | 4 | 4 | 3 | 5 | 4 | 3 | 4 | 5 | 4 | 3 | 2 | 4 |
| 171 | 4 | 5 | 4 | 5 | 3 | 3 | 4 | 5 | 5 | 5 | 3 | 5 |
| 172 | 5 | 5 | 3 | 3 | 4 | 3 | 4 | 3 | 3 | 4 | 3 | 4 |
| 173 | 5 | 4 | 3 | 5 | 5 | 4 | 4 | 5 | 5 | 4 | 4 | 4 |
| 174 | 3 | 4 | 4 | 4 | 4 | 3 | 5 | 5 | 4 | 3 | 3 | 5 |
| 175 | 4 | 5 | 4 | 5 | 5 | 4 | 4 | 4 | 4 | 4 | 3 | 5 |
| 176 | 4 | 4 | 5 | 5 | 3 | 2 | 4 | 4 | 3 | 2 | 4 | 3 |
| 177 | 4 | 3 | 5 | 5 | 3 | 3 | 5 | 5 | 4 | 5 | 2 | 4 |
| 178 | 4 | 5 | 5 | 5 | 4 | 4 | 4 | 5 | 5 | 5 | 4 | 5 |
| 179 | 4 | 4 | 3 | 1 | 5 | 4 | 3 | 4 | 5 | 4 | 3 | 3 |
| 180 | 5 | 4 | 4 | 4 | 4 | 4 | 5 | 4 | 3 | 4 | 4 | 5 |
| 181 | 5 | 5 | 4 | 3 | 2 | 2 | 4 | 2 | 1 | 1 | 4 | 3 |
| 184 | 3 | 4 | 4 | 4 | 3 | 3 | 3 | 3 | 4 | 3 | 1 | 4 |
| 185 | 3 | 5 | 4 | 5 | 4 | 4 | 4 | 5 | 4 | 4 | 3 | 5 |
| 186 | 5 | 4 | 4 | 4 | 4 | 4 | 4 | 4 | 5 | 4 | 4 | 4 |
| 187 | 5 | 5 | 5 | 5 | 5 | 5 | 5 | 5 | 5 | 5 | 5 | 5 |
| 188 | 5 | 2 | 5 | 5 | 4 | 4 | 4 | 5 | 5 |  | 5 | 5 |
| 189 | 3 | 5 | 5 | 4 | 5 | 4 | 5 | 4 | 5 | 4 | 4 | 5 |
| 190 | 4 | 5 | 4 | 5 | 5 | 3 | 5 | 5 | 4 | 4 | 5 | 5 |
| 192 | 5 | 5 | 4 | 5 | 4 | 5 | 4 | 5 | 5 | 5 | 5 | 5 |
| 194 | 5 | 5 | 4 | 4 | 4 | 4 | 5 | 5 | 4 | 4 | 3 | 4 |
| 195 | 4 | 4 | 4 | 4 | 4 | 4 | 4 | 4 | 4 | 4 | 4 | 4 |
| 200 | 4 | 5 | 5 | 5 | 4 | 5 | 5 | 5 | 4 | 5 | 3 | 4 |
| 210 | 3 | 4 | 5 | 4 | 4 | 5 | 4 | 4 | 4 | 3 | 3 | 4 |
| 211 | 2 | 4 | 5 | 5 | 4 | 1 | 4 | 5 | 4 | 4 | 3 | 5 |

**Key:**

2.1.a) = You receive adequate academic study opportunities from your organisation to promote innovation.

2.1.b) = You have the relevant academic qualifications to help drive agriculture innovation.

2.1.c) = You need additional academic qualifications to improve your innovativeness

2.1.d) = Improving learning will significantly improve innovation within your organisation.

2.2.a) = Training programs within your organisation are effective in enhancing innovation

2.2.b) = Your organisation HR supports you to attend relevant training opportunities.

2.2.c) = You would require further training to be more innovative and productive.

2.2.d) = Improving training will significantly improve innovation within your organisation.

2.3.a) = Your direct supervisor offers you adequate guidance to stimulate innovation whenever needed.

2.3.b) = Coaching has helped improve your innovativeness within your organisation.

2.3.c) = You receive adequate coaching from external coaches from outside your organisation.

2.3.d) = Improving coaching will significantly improve innovation within your organisation.

5 = Strongly agree; 4 = Agree; 3 = Neutral; 2 = Disagree; 1 = Strongly disagree

## Appendix 4. Raw data on variables related to challenges and strategies to improve delivery of capacity building in your organisation.

| ID | 3a | 3b | 3c | 3d | 3e | 3f | 3g | 3h | 4a | 4b | 4c | 4d | 4e | 4f | 4g | 4h | 4i | 5a | 5b | 5c | 5d | 5e | 5f | 5g | 5h |
| --- | --- | --- | --- | --- | --- | --- | --- | --- | --- | --- | --- | --- | --- | --- | --- | --- | --- | --- | --- | --- | --- | --- | --- | --- | --- |
| 3 | 0 | 0 | 1 | 0 | 0 | 0 | 0 | 0 | 0 | 0 | 0 | 0 | 0 | 1 | 0 | 0 | 0 | 1 | 1 | 1 | 1 | 1 | 1 | 1 | 1 |
| 4 | 0 | 0 | 0 | 0 | 0 | 1 | 1 | 1 | 1 | 1 | 1 | 1 | 1 | 1 | 0 | 0 | 0 | 0 | 1 | 1 | 0 | 0 | 1 | 1 | 1 |
| 6 | 1 | 1 | 1 | 1 | 1 | 0 | 1 | 1 | 0 | 1 | 1 | 1 | 1 | 1 | 1 | 1 | 0 | 0 | 1 | 0 | 1 | 1 | 1 | 1 | 1 |
| 7 | 0 | 0 | 1 | 0 | 0 | 1 | 0 | 0 | 0 | 0 | 1 | 0 | 0 | 1 | 0 | 0 | 0 | 0 | 1 | 0 | 0 | 1 | 1 | 0 | 1 |
| 10 | 0 | 0 | 1 | 0 | 0 | 0 | 0 | 0 | 0 | 0 | 0 | 0 | 0 | 1 | 0 | 0 | 0 | 0 | 0 | 0 | 0 | 0 | 1 | 0 | 0 |
| 11 | 0 | 0 | 1 | 0 | 0 | 0 | 0 | 0 | 0 | 0 | 1 | 0 | 0 | 0 | 0 | 0 | 0 | 0 | 1 | 0 | 0 | 0 | 0 | 0 | 0 |
| 12 | 0 | 0 | 1 | 0 | 1 | 0 | 0 | 0 | 1 | 0 | 0 | 0 | 0 | 0 | 0 | 1 | 0 | 1 | 0 | 0 | 1 | 0 | 0 | 0 | 0 |
| 14 | 1 | 0 | 1 | 0 | 0 | 1 | 1 | 0 | 1 | 1 | 1 | 1 | 1 | 1 | 1 | 1 | 1 | 0 | 1 | 0 | 1 | 0 | 1 | 0 | 1 |
| 18 | 0 | 0 | 1 | 0 | 0 | 0 | 0 | 0 | 0 | 0 | 1 | 0 | 0 | 0 | 0 | 0 | 0 | 0 | 1 | 0 | 0 | 1 | 1 | 0 | 0 |
| 19 | 1 | 1 | 1 | 1 | 1 | 1 | 1 | 1 | 1 | 1 | 1 | 1 | 1 | 1 | 1 | 1 | 1 | 1 | 1 | 1 | 1 | 1 | 1 | 1 | 1 |
| 20 | 0 | 0 | 1 | 0 | 0 | 0 | 0 | 1 | 0 | 0 | 0 | 0 | 0 | 1 | 0 | 1 | 0 | 0 | 1 | 1 | 0 | 1 | 1 | 0 | 1 |
| 22 | 0 | 1 | 1 | 0 | 0 | 0 | 1 | 0 | 0 | 0 | 1 | 0 | 0 | 0 | 1 | 1 | 0 | 1 | 1 | 1 | 1 | 1 | 1 | 1 | 1 |
| 24 | 0 | 0 | 1 | 1 | 0 | 0 | 1 | 1 | 1 | 1 | 1 | 0 | 1 | 1 | 1 | 0 | 1 | 1 | 1 | 0 | 0 | 1 | 1 | 0 | 1 |
| 25 | 0 | 0 | 1 | 0 | 0 | 1 | 1 | 0 | 0 | 1 | 1 | 0 | 0 | 1 | 0 | 1 | 0 | 0 | 1 | 0 | 1 | 1 | 1 | 0 | 1 |
| 26 | 0 | 0 | 1 | 0 | 0 | 0 | 0 | 0 | 0 | 0 | 1 | 0 | 0 | 0 | 0 | 1 | 0 | 0 | 1 | 0 | 0 | 1 | 0 | 0 | 0 |
| 27 | 0 | 1 | 1 | 1 | 0 | 1 | 1 | 1 | 1 | 1 | 1 | 1 | 1 | 1 | 0 | 1 | 1 | 0 | 1 | 1 | 0 | 0 | 1 | 1 | 1 |
| 28 | 0 | 1 | 1 | 1 | 1 | 1 | 1 | 0 | 1 | 0 | 1 | 0 | 0 | 1 | 1 | 1 | 0 | 1 | 1 | 0 | 1 | 1 | 1 | 0 | 1 |
| 29 | 0 | 0 | 0 | 0 | 1 | 0 | 1 | 0 | 0 | 1 | 1 | 0 | 0 | 1 | 0 | 0 | 0 | 0 | 0 | 0 | 0 | 1 | 0 | 0 | 0 |
| 31 | 0 | 1 | 1 | 0 | 1 | 0 | 1 | 0 | 1 | 0 | 1 | 0 | 1 | 1 | 0 | 0 | 0 | 0 | 0 | 1 | 0 | 1 | 1 | 0 | 1 |
| 32 | 1 | 0 | 1 | 1 | 1 | 0 | 0 | 1 | 1 | 0 | 1 | 0 | 0 | 1 | 0 | 0 | 1 | 1 | 1 | 0 | 0 | 1 | 0 | 0 | 1 |
| 33 | 0 | 0 | 1 | 0 | 1 | 0 | 0 | 0 | 1 | 1 | 1 | 1 | 1 | 1 | 1 | 1 | 1 | 1 | 1 | 1 | 1 | 1 | 1 | 1 | 1 |
| 34 | 0 | 1 | 1 | 1 | 1 | 1 | 1 | 0 | 0 | 1 | 1 | 0 | 1 | 1 | 1 | 1 | 1 | 1 | 1 | 0 | 1 | 1 | 1 | 1 | 1 |
| 36 | 0 | 1 | 1 | 0 | 0 | 0 | 0 | 0 | 0 | 1 | 1 | 1 | 0 | 1 | 0 | 0 | 0 | 1 | 1 | 0 | 1 | 1 | 0 | 1 | 0 |
| 37 | 1 | 0 | 1 | 0 | 1 | 1 | 1 | 0 | 0 | 0 | 1 | 0 | 1 | 1 | 0 | 1 | 0 | 0 | 1 | 0 | 1 | 0 | 1 | 0 | 1 |
| 38 | 0 | 1 | 1 | 0 | 1 | 1 | 1 | 0 | 1 | 1 | 1 | 1 | 1 | 1 | 1 | 1 | 1 | 0 | 1 | 1 | 1 | 1 | 1 | 0 | 1 |
| 39 | 1 | 1 | 1 | 1 | 1 | 0 | 1 | 0 | 0 | 1 | 1 | 1 | 1 | 1 | 1 | 1 | 1 | 0 | 1 | 0 | 0 | 1 | 1 | 1 | 1 |
| 40 | 0 | 1 | 1 | 1 | 0 | 1 | 1 | 0 | 1 | 1 | 1 | 0 | 0 | 1 | 1 | 1 | 1 | 0 | 1 | 0 | 0 | 1 | 1 | 1 | 1 |
| 41 | 0 | 0 | 0 | 0 | 1 | 1 | 1 | 0 | 1 | 1 | 1 | 1 | 1 | 1 | 1 | 1 | 1 | 0 | 1 | 0 | 1 | 1 | 1 | 1 | 1 |
| 42 | 0 | 0 | 0 | 1 | 1 | 1 | 1 | 0 | 1 | 1 | 1 | 1 | 1 | 1 | 1 | 1 | 1 | 0 | 1 | 0 | 1 | 1 | 1 | 1 | 1 |
| 43 | 0 | 0 | 0 | 1 | 1 | 1 | 1 | 0 | 1 | 1 | 1 | 1 | 1 | 1 | 1 | 1 | 1 | 0 | 1 | 0 | 1 | 1 | 1 | 1 | 1 |
| 44 | 0 | 0 | 0 | 1 | 1 | 1 | 1 | 0 | 1 | 1 | 1 | 1 | 1 | 1 | 1 | 1 | 1 | 0 | 1 | 0 | 1 | 1 | 1 | 1 | 1 |
| 46 | 0 | 1 | 1 | 1 | 1 | 1 | 1 | 1 | 1 | 1 | 1 | 1 | 1 | 1 | 1 | 1 | 1 | 0 | 1 | 0 | 1 | 1 | 1 | 1 | 1 |
| 50 | 1 | 1 | 1 | 1 | 0 | 0 | 1 | 1 | 0 | 1 | 1 | 1 | 1 | 1 | 1 | 1 | 0 | 1 | 1 | 1 | 1 | 1 | 1 | 1 | 0 |
| 51 | 1 | 1 | 1 | 1 | 1 | 0 | 0 | 0 | 1 | 1 | 1 | 1 | 0 | 1 | 1 | 0 | 0 | 1 | 0 | 0 | 0 | 0 | 0 | 1 | 0 |
| 52 | 0 | 0 | 0 | 0 | 1 | 0 | 1 | 0 | 0 | 0 | 1 | 0 | 0 | 0 | 0 | 1 | 0 | 0 | 0 | 0 | 0 | 1 | 0 | 0 | 1 |
| 53 | 0 | 0 | 1 | 0 | 0 | 0 | 0 | 0 | 1 | 0 | 1 | 0 | 0 | 0 | 0 | 0 | 0 | 0 | 1 | 0 | 0 | 0 | 1 | 1 | 0 |
| 54 | 0 | 1 | 1 | 0 | 1 | 0 | 1 | 1 | 0 | 1 | 1 | 0 | 1 | 1 | 1 | 1 | 0 | 0 | 1 | 1 | 1 | 1 | 1 | 0 | 1 |
| 55 | 0 | 0 | 1 | 0 | 0 | 1 | 0 | 1 | 1 | 0 | 0 | 1 | 0 | 1 | 0 | 0 | 0 | 1 | 0 | 0 | 1 | 0 | 0 | 0 | 1 |
| 56 | 0 | 0 | 1 | 0 | 1 | 1 | 0 | 0 | 1 | 1 | 1 | 1 | 1 | 1 | 1 | 1 | 1 | 0 | 1 | 0 | 0 | 1 | 1 | 1 | 1 |
| 57 | 1 | 1 | 1 | 1 | 1 | 1 | 1 | 1 | 1 | 1 | 1 | 1 | 1 | 1 | 1 | 1 | 1 | 0 | 0 | 1 | 0 | 0 | 1 | 1 | 1 |
| 58 | 1 | 0 | 1 | 0 | 1 | 0 | 1 | 0 | 0 | 0 | 1 | 0 | 1 | 0 | 0 | 1 | 1 | 0 | 0 | 1 | 0 | 1 | 0 | 1 | 1 |
| 59 | 0 | 0 | 1 | 0 | 0 | 0 | 0 | 0 | 1 | 0 | 0 | 0 | 0 | 0 | 0 | 0 | 0 | 0 | 1 | 0 | 0 | 0 | 0 | 0 | 0 |
| 60 | 1 | 1 | 1 | 1 | 0 | 0 | 0 | 0 | 1 | 1 | 1 | 0 | 1 | 0 | 1 | 1 | 1 | 0 | 1 | 0 | 1 | 1 | 1 | 1 | 1 |
| 61 | 1 | 1 | 1 | 0 | 0 | 0 | 0 | 0 | 1 | 1 | 1 | 1 | 1 | 1 | 1 | 1 | 1 | 0 | 1 | 1 | 1 | 1 | 1 | 1 | 1 |
| 62 | 1 | 0 | 1 | 1 | 0 | 0 | 1 | 1 | 1 | 1 | 1 | 0 | 1 | 1 | 0 | 1 | 1 | 1 | 1 | 1 | 1 | 1 | 0 | 1 | 1 |
| 63 | 0 | 1 | 0 | 1 | 1 | 0 | 1 | 1 | 1 | 1 | 1 | 0 | 1 | 1 | 1 | 0 | 1 | 1 | 1 | 1 | 1 | 1 | 1 | 0 | 1 |
| 65 | 1 | 1 | 1 | 1 | 1 | 0 | 0 | 0 | 0 | 1 | 1 | 0 | 1 | 1 | 0 | 0 | 1 | 0 | 1 | 0 | 1 | 0 | 1 | 0 | 1 |
| 66 | 1 | 1 | 1 | 1 | 1 | 0 | 1 | 0 | 1 | 1 | 1 | 1 | 0 | 1 | 1 | 1 | 1 | 0 | 1 | 1 | 1 | 1 | 1 | 1 | 1 |
| 67 | 0 | 0 | 1 | 0 | 0 | 0 | 0 | 0 | 0 | 0 | 1 | 0 | 0 | 0 | 0 | 0 | 0 | 0 | 1 | 0 | 0 | 0 | 0 | 0 | 0 |
| 68 | 1 | 1 | 1 | 0 | 0 | 1 | 0 | 0 | 1 | 1 | 1 | 1 | 1 | 1 | 1 | 1 | 1 | 0 | 1 | 1 | 1 | 1 | 1 | 1 | 1 |
| 69 | 0 | 1 | 1 | 0 | 0 | 0 | 1 | 1 | 1 | 1 | 1 | 1 | 1 | 1 | 1 | 1 | 1 | 0 | 1 | 0 | 1 | 1 | 0 | 1 | 1 |
| 70 | 0 | 0 | 0 | 0 | 0 | 1 | 0 | 0 | 0 | 1 | 0 | 0 | 0 | 0 | 0 | 0 | 0 | 0 | 1 | 0 | 0 | 0 | 0 | 0 | 0 |
| 71 | 1 | 0 | 1 | 0 | 0 | 1 | 0 | 1 | 1 | 1 | 1 | 1 | 0 | 0 | 1 | 1 | 1 | 0 | 1 | 1 | 0 | 0 | 1 | 1 | 0 |
| 72 | 0 | 0 | 1 | 0 | 0 | 0 | 0 | 0 | 0 | 0 | 0 | 0 | 0 | 0 | 0 | 1 | 0 | 1 | 0 | 0 | 0 | 0 | 0 | 0 | 0 |
| 73 | 0 | 1 | 1 | 0 | 0 | 0 | 0 | 0 | 0 | 0 | 1 | 0 | 0 | 0 | 0 | 1 | 0 | 0 | 1 | 0 | 0 | 1 | 0 | 1 | 1 |
| 74 | 0 | 1 | 1 | 1 | 1 | 0 | 1 | 1 | 1 | 0 | 1 | 0 | 1 | 1 | 1 | 1 | 1 | 1 | 1 | 1 | 1 | 1 | 1 | 1 | 1 |
| 75 | 0 | 1 | 1 | 1 | 1 | 1 | 1 | 1 | 1 | 1 | 1 | 1 | 1 | 1 | 1 | 1 | 1 | 1 | 1 | 1 | 1 | 1 | 1 | 1 | 1 |
| 77 | 1 | 0 | 1 | 1 | 0 | 0 | 1 | 0 | 1 | 0 | 1 | 0 | 1 | 1 | 1 | 0 | 1 | 1 | 0 | 1 | 1 | 0 | 1 | 0 | 1 |
| 80 | 1 | 1 | 1 | 1 | 1 | 1 | 1 | 1 | 1 | 1 | 1 | 1 | 1 | 1 | 1 | 1 | 1 | 1 | 1 | 1 | 1 | 1 | 1 | 1 | 1 |
| 81 | 0 | 0 | 1 | 1 | 1 | 1 | 0 | 0 | 1 | 1 | 1 | 1 | 0 | 1 | 1 | 0 | 0 | 0 | 1 | 0 | 1 | 1 | 0 | 0 | 1 |
| 82 | 0 | 0 | 0 | 0 | 0 | 0 | 1 | 0 | 0 | 0 | 0 | 0 | 0 | 1 | 0 | 0 | 0 | 0 | 0 | 0 | 0 | 0 | 0 | 0 | 1 |
| 84 | 0 | 0 | 1 | 0 | 0 | 0 | 0 | 0 | 1 | 1 | 1 | 1 | 1 | 1 | 1 | 1 | 1 | 1 | 0 | 0 | 0 | 0 | 0 | 0 | 0 |
| 86 | 1 | 1 | 1 | 0 | 1 | 1 | 1 | 1 | 1 | 1 | 1 | 0 | 0 | 1 | 1 | 1 | 1 | 1 | 0 | 1 | 1 | 1 | 1 | 1 | 1 |
| 86 | 0 | 1 | 1 | 0 | 0 | 0 | 0 | 0 | 1 | 1 | 1 | 1 | 1 | 1 | 1 | 1 | 1 | 1 | 1 | 1 | 1 | 1 | 1 | 1 | 1 |
| 88 | 1 | 1 | 1 | 1 | 1 | 1 | 1 | 1 | 1 | 1 | 1 | 0 | 0 | 0 | 1 | 1 | 0 | 1 | 1 | 1 | 1 | 1 | 1 | 1 | 1 |
| 89 | 1 | 1 | 1 | 1 | 0 | 0 | 0 | 0 | 1 | 1 | 1 | 0 | 0 | 0 | 0 | 0 | 0 | 0 | 1 | 0 | 1 | 0 | 1 | 0 | 1 |
| 91 | 1 | 1 | 1 | 0 | 1 | 1 | 1 | 0 | 1 | 1 | 1 | 0 | 0 | 1 | 1 | 1 | 1 | 0 | 1 | 1 | 1 | 1 | 1 | 1 | 1 |
| 93 | 0 | 1 | 1 | 0 | 0 | 0 | 1 | 0 | 1 | 1 | 0 | 0 | 0 | 0 | 0 | 1 | 0 | 0 | 1 | 0 | 0 | 0 | 0 | 1 | 1 |
| 94 | 0 | 0 | 1 | 0 | 0 | 0 | 1 | 0 | 1 | 0 | 1 | 0 | 1 | 1 | 0 | 0 | 1 | 1 | 1 | 0 | 0 | 1 | 1 | 1 | 1 |
| 95 | 1 | 1 | 1 | 0 | 1 | 0 | 1 | 1 | 0 | 1 | 1 | 0 | 1 | 0 | 1 | 1 | 0 | 1 | 1 | 0 | 1 | 0 | 1 | 0 | 1 |
| 96 | 1 | 1 | 1 | 0 | 0 | 0 | 1 | 1 | 0 | 0 | 1 | 1 | 1 | 1 | 1 | 0 | 0 | 1 | 0 | 0 | 0 | 1 | 1 | 1 | 1 |
| 98 | 0 | 0 | 1 | 0 | 0 | 0 | 0 | 0 | 1 | 0 | 0 | 0 | 1 | 0 | 0 | 1 | 0 | 0 | 1 | 0 | 0 | 1 | 0 | 0 | 1 |
| 103 | 0 | 0 | 1 | 1 | 1 | 1 | 1 | 1 | 1 | 1 | 1 | 1 | 1 | 1 | 1 | 1 | 1 | 1 | 1 | 1 | 1 | 1 | 1 | 0 | 1 |
| 105 | 0 | 1 | 1 | 1 | 0 | 0 | 0 | 0 | 0 | 0 | 1 | 0 | 0 | 1 | 0 | 0 | 1 | 0 | 1 | 0 | 0 | 1 | 1 | 0 | 1 |
| 106 | 1 | 0 | 1 | 0 | 1 | 0 | 1 | 0 | 1 | 1 | 0 | 0 | 1 | 1 | 1 | 1 | 0 | 0 | 0 | 0 | 1 | 1 | 1 | 1 | 1 |
| 107 | 0 | 0 | 0 | 0 | 0 | 0 | 0 | 0 | 0 | 0 | 0 | 0 | 0 | 1 | 0 | 1 | 0 | 0 | 1 | 0 | 0 | 0 | 0 | 0 | 1 |
| 108 | 1 | 1 | 1 | 1 | 1 | 1 | 1 | 1 | 1 | 1 | 1 | 1 | 1 | 1 | 1 | 1 | 1 | 0 | 1 | 1 | 1 | 1 | 1 | 1 | 1 |
| 109 | 1 | 1 | 1 | 1 | 1 | 1 | 1 | 0 | 1 | 1 | 1 | 1 | 1 | 1 | 1 | 1 | 1 | 1 | 1 | 1 | 1 | 1 | 1 | 1 | 1 |
| 109 | 0 | 0 | 1 | 0 | 0 | 1 | 0 | 1 | 0 | 0 | 1 | 0 | 0 | 1 | 0 | 1 | 0 | 0 | 1 | 1 | 0 | 0 | 1 | 1 | 1 |
| 110 | 0 | 0 | 1 | 1 | 1 | 1 | 1 | 1 | 1 | 1 | 1 | 1 | 1 | 1 | 1 | 1 | 1 | 1 | 1 | 1 | 1 | 1 | 1 | 1 | 1 |
| 111 | 0 | 0 | 1 | 1 | 1 | 1 | 1 | 1 | 1 | 1 | 1 | 1 | 1 | 1 | 1 | 1 | 1 | 0 | 1 | 1 | 1 | 1 | 1 | 0 | 1 |
| 112 | 0 | 0 | 1 | 1 | 0 | 1 | 0 | 0 | 1 | 1 | 1 | 0 | 1 | 1 | 1 | 1 | 0 | 1 | 1 | 0 | 1 | 0 | 1 | 1 | 1 |
| 116 | 0 | 1 | 1 | 1 | 1 | 1 | 1 | 1 | 1 | 1 | 1 | 1 | 1 | 1 | 1 | 1 | 1 | 0 | 1 | 1 | 1 | 1 | 1 | 1 | 1 |
| 117 | 1 | 1 | 0 | 1 | 1 | 1 | 1 | 1 | 1 | 1 | 1 | 1 | 1 | 1 | 1 | 1 | 1 | 1 | 1 | 1 | 1 | 1 | 1 | 1 | 1 |
| 118 | 0 | 0 | 1 | 0 | 0 | 0 | 0 | 0 | 0 | 1 | 1 | 1 | 0 | 0 | 0 | 1 | 1 | 1 | 1 | 0 | 0 | 0 | 1 | 0 | 1 |
| 119 | 1 | 0 | 1 | 0 | 0 | 1 | 0 | 0 | 1 | 1 | 1 | 0 | 0 | 0 | 1 | 0 | 0 | 1 | 0 | 0 | 0 | 1 | 0 | 1 | 0 |
| 120 | 0 | 1 | 1 | 1 | 1 | 1 | 1 | 1 | 0 | 1 | 1 | 1 | 1 | 1 | 1 | 1 | 1 | 1 | 1 | 1 | 1 | 1 | 1 | 1 | 1 |
| 121 | 0 | 1 | 1 | 1 | 1 | 1 | 1 | 1 | 1 | 1 | 1 | 1 | 1 | 1 | 1 | 1 | 1 | 1 | 1 | 1 | 1 | 1 | 1 | 1 | 1 |
| 122 | 1 | 1 | 1 | 1 | 1 | 1 | 1 | 1 | 1 | 1 | 1 | 1 | 1 | 1 | 1 | 1 | 1 | 1 | 1 | 1 | 1 | 1 | 1 | 1 | 1 |
| 152 | 0 | 1 | 1 | 0 | 1 | 0 | 1 | 0 | 1 | 1 | 1 | 1 | 0 | 1 | 1 | 1 | 0 | 1 | 1 | 0 | 0 | 1 | 1 | 0 | 1 |
| 154 | 0 | 0 | 1 | 1 | 0 | 1 | 1 | 1 | 1 | 0 | 1 | 0 | 1 | 1 | 1 | 1 | 0 | 1 | 1 | 1 | 1 | 1 | 0 | 1 | 1 |
| 153 | 0 | 0 | 1 | 0 | 1 | 0 | 0 | 0 | 0 | 1 | 1 | 0 | 0 | 1 | 0 | 1 | 1 | 1 | 1 | 0 | 0 | 1 | 1 | 0 | 1 |
| 155 | 0 | 0 | 1 | 0 | 0 | 0 | 0 | 1 | 1 | 0 | 1 | 0 | 0 | 0 | 0 | 1 | 0 | 0 | 1 | 0 | 0 | 0 | 1 | 0 | 1 |
| 156 | 0 | 0 | 1 | 0 | 1 | 0 | 0 | 0 | 0 | 0 | 1 | 0 | 0 | 1 | 0 | 0 | 0 | 0 | 0 | 1 | 0 | 0 | 1 | 0 | 0 |
| 157 | 0 | 0 | 1 | 0 | 0 | 1 | 1 | 1 | 1 | 0 | 1 | 0 | 0 | 1 | 0 | 1 | 0 | 0 | 1 | 1 | 0 | 1 | 1 | 1 | 1 |
| 158 | 1 | 0 | 1 | 1 | 1 | 1 | 1 | 0 | 1 | 1 | 1 | 1 | 1 | 1 | 1 | 1 | 1 | 1 | 1 | 1 | 1 | 1 | 1 | 1 | 1 |
| 159 | 0 | 0 | 1 | 1 | 1 | 1 | 1 | 0 | 1 | 1 | 1 | 1 | 1 | 1 | 1 | 1 | 0 | 0 | 0 | 1 | 1 | 1 | 1 | 0 | 1 |
| 161 | 1 | 1 | 1 | 1 | 1 | 0 | 1 | 1 | 1 | 1 | 1 | 1 | 1 | 1 | 1 | 1 | 1 | 1 | 1 | 1 | 1 | 1 | 1 | 1 | 1 |
| 162 | 0 | 0 | 1 | 0 | 1 | 0 | 1 | 1 | 0 | 0 | 1 | 0 | 1 | 0 | 1 | 1 | 1 | 1 | 1 | 0 | 0 | 0 | 0 | 0 | 1 |
| 163 | 0 | 0 | 1 | 0 | 0 | 1 | 1 | 0 | 0 | 0 | 1 | 0 | 0 | 1 | 0 | 1 | 0 | 0 | 1 | 1 | 0 | 1 | 0 | 0 | 1 |
| 164 | 0 | 0 | 1 | 0 | 0 | 0 | 0 | 0 | 0 | 0 | 1 | 0 | 0 | 1 | 0 | 0 | 0 | 0 | 0 | 0 | 0 | 1 | 0 | 0 | 1 |
| 166 | 1 | 0 | 1 | 1 | 1 | 1 | 1 | 1 | 1 | 1 | 1 | 1 | 1 | 1 | 1 | 1 | 1 | 0 | 1 | 1 | 1 | 1 | 1 | 1 | 1 |
| 167 | 0 | 0 | 1 | 0 | 0 | 1 | 0 | 0 | 1 | 0 | 0 | 0 | 0 | 1 | 0 | 0 | 0 | 0 | 0 | 0 | 0 | 0 | 0 | 0 | 1 |
| 168 | 0 | 0 | 1 | 0 | 0 | 1 | 0 | 0 | 1 | 0 | 0 | 0 | 1 | 0 | 0 | 0 | 0 | 0 | 0 | 0 | 0 | 1 | 1 | 0 | 1 |
| 169 | 0 | 0 | 1 | 1 | 1 | 1 | 1 | 1 | 1 | 0 | 1 | 1 | 0 | 0 | 1 | 1 | 0 | 0 | 1 | 0 | 1 | 1 | 1 | 1 | 1 |
| 170 | 0 | 0 | 1 | 0 | 0 | 1 | 1 | 1 | 1 | 0 | 0 | 0 | 0 | 1 | 1 | 1 | 0 | 0 | 1 | 1 | 0 | 1 | 1 | 1 | 0 |
| 171 | 0 | 0 | 1 | 0 | 1 | 1 | 0 | 0 | 1 | 1 | 1 | 0 | 0 | 1 | 1 | 0 | 1 | 1 | 1 | 1 | 1 | 1 | 1 | 1 | 1 |
| 172 | 0 | 0 | 1 | 1 | 0 | 0 | 1 | 0 | 0 | 0 | 1 | 0 | 1 | 1 | 0 | 0 | 0 | 0 | 1 | 0 | 1 | 0 | 0 | 0 | 1 |
| 173 | 0 | 1 | 0 | 0 | 1 | 0 | 0 | 0 | 0 | 0 | 1 | 0 | 0 | 1 | 0 | 1 | 0 | 0 | 0 | 0 | 0 | 1 | 0 | 1 | 1 |
| 174 | 0 | 0 | 1 | 0 | 0 | 1 | 0 | 0 | 1 | 1 | 0 | 0 | 0 | 1 | 0 | 1 | 1 | 1 | 1 | 1 | 0 | 1 | 1 | 1 | 1 |
| 175 | 0 | 0 | 1 | 0 | 1 | 0 | 0 | 0 | 0 | 0 | 1 | 0 | 0 | 0 | 0 | 1 | 0 | 0 | 1 | 0 | 0 | 0 | 1 | 0 | 0 |
| 176 | 0 | 0 | 1 | 0 | 0 | 0 | 1 | 0 | 0 | 0 | 0 | 0 | 0 | 1 | 0 | 1 | 0 | 0 | 1 | 0 | 0 | 1 | 0 | 0 | 1 |
| 177 | 1 | 1 | 1 | 0 | 1 | 0 | 1 | 0 | 1 | 0 | 1 | 0 | 0 | 1 | 0 | 0 | 1 | 1 | 1 | 0 | 0 | 1 | 1 | 0 | 1 |
| 178 | 1 | 0 | 1 | 0 | 0 | 0 | 1 | 0 | 0 | 1 | 1 | 1 | 0 | 0 | 1 | 1 | 0 | 0 | 0 | 0 | 0 | 1 | 1 | 1 | 1 |
| 179 | 0 | 0 | 1 | 0 | 0 | 1 | 0 | 0 | 0 | 0 | 1 | 0 | 1 | 0 | 0 | 1 | 0 | 0 | 0 | 1 | 0 | 1 | 0 | 1 | 0 |
| 180 | 0 | 0 | 1 | 1 | 0 | 0 | 1 | 0 | 0 | 0 | 1 | 0 | 0 | 1 | 1 | 1 | 0 | 0 | 1 | 0 | 0 | 1 | 1 | 1 | 1 |
| 181 | 1 | 0 | 1 | 1 | 0 | 1 | 0 | 1 | 0 | 1 | 1 | 0 | 0 | 1 | 1 | 1 | 1 | 0 | 1 | 0 | 1 | 1 | 1 | 0 | 1 |
| 184 | 0 | 0 | 1 | 0 | 0 | 0 | 0 | 0 | 0 | 0 | 1 | 0 | 0 | 1 | 0 | 0 | 0 | 0 | 1 | 0 | 0 | 0 | 0 | 0 | 0 |
| 185 | 0 | 0 | 1 | 1 | 1 | 0 | 1 | 0 | 0 | 1 | 1 | 1 | 1 | 1 | 1 | 1 | 1 | 0 | 1 | 1 | 0 | 0 | 1 | 0 | 1 |
| 186 | 0 | 0 | 1 | 0 | 1 | 0 | 1 | 0 | 0 | 1 | 1 | 0 | 0 | 1 | 0 | 1 | 1 | 1 | 1 | 1 | 0 | 0 | 1 | 0 | 0 |
| 187 | 1 | 1 | 1 | 0 | 0 | 0 | 0 | 0 | 0 | 1 | 1 | 1 | 0 | 1 | 1 | 1 | 1 | 0 | 1 | 1 | 0 | 1 | 0 | 0 | 1 |
| 188 | 0 | 1 | 1 | 0 | 1 | 0 | 1 | 1 | 0 | 1 | 1 | 1 | 1 | 1 | 1 | 0 | 0 | 1 | 1 | 1 | 1 | 1 | 1 | 1 | 1 |
| 189 | 1 | 1 | 1 | 1 | 0 | 0 | 0 | 0 | 1 | 1 | 1 | 1 | 1 | 1 | 1 | 1 | 1 | 1 | 1 | 1 | 1 | 1 | 1 | 1 | 1 |
| 190 | 0 | 1 | 1 | 1 | 1 | 1 | 1 | 1 | 0 | 0 | 1 | 1 | 1 | 1 | 1 | 1 | 0 | 0 | 1 | 1 | 1 | 1 | 0 | 1 | 1 |
| 192 | 0 | 0 | 1 | 0 | 1 | 0 | 0 | 0 | 0 | 0 | 0 | 0 | 1 | 1 | 0 | 0 | 0 | 0 | 1 | 0 | 1 | 0 | 1 | 0 | 1 |
| 194 | 0 | 1 | 1 | 1 | 1 | 0 | 1 | 0 | 1 | 0 | 1 | 1 | 1 | 1 | 0 | 1 | 0 | 0 | 0 | 0 | 1 | 1 | 1 | 1 | 1 |
| 195 | 0 | 0 | 1 | 0 | 0 | 0 | 0 | 1 | 0 | 1 | 1 | 0 | 1 | 1 | 0 | 0 | 0 | 0 | 1 | 0 | 0 | 0 | 1 | 0 | 1 |
| 200 | 0 | 0 | 1 | 0 | 0 | 1 | 1 | 0 | 1 | 1 | 1 | 1 | 1 | 1 | 1 | 1 | 1 | 1 | 1 | 1 | 0 | 1 | 1 | 1 | 1 |
| 200 | 1 | 1 | 1 | 0 | 1 | 0 | 1 | 1 | 0 | 1 | 1 | 0 | 1 | 1 | 1 | 1 | 1 | 0 | 1 | 1 | 0 | 1 | 1 | 1 | 1 |
| 201 | 0 | 0 | 1 | 0 | 0 | 0 | 1 | 0 | 0 | 1 | 1 | 0 | 0 | 1 | 1 | 1 | 1 | 0 | 1 | 0 | 0 | 1 | 0 | 0 | 1 |
| 202 | 1 | 1 | 1 | 1 | 1 | 1 | 1 | 1 | 1 | 1 | 1 | 1 | 1 | 1 | 1 | 1 | 1 | 1 | 1 | 1 | 1 | 1 | 1 | 1 | 1 |
| 203 | 0 | 0 | 1 | 0 | 0 | 1 | 1 | 0 | 1 | 0 | 1 | 0 | 0 | 0 | 0 | 1 | 0 | 0 | 1 | 0 | 0 | 1 | 0 | 1 | 1 |
| 204 | 0 | 1 | 1 | 0 | 1 | 0 | 1 | 1 | 0 | 1 | 1 | 0 | 1 | 1 | 1 | 1 | 1 | 0 | 1 | 1 | 1 | 1 | 1 | 1 | 1 |
| 205 | 1 | 0 | 1 | 0 | 1 | 0 | 1 | 1 | 0 | 0 | 1 | 0 | 1 | 1 | 1 | 1 | 0 | 1 | 1 | 1 | 0 | 1 | 1 | 0 | 0 |
| 206 | 0 | 0 | 0 | 0 | 0 | 0 | 0 | 0 | 0 | 0 | 0 | 0 | 0 | 0 | 0 | 0 | 0 | 0 | 0 | 0 | 0 | 0 | 0 | 0 | 0 |
| 207 | 1 | 0 | 1 | 0 | 0 | 0 | 1 | 0 | 0 | 1 | 1 | 0 | 0 | 1 | 0 | 1 | 0 | 0 | 0 | 1 | 1 | 1 | 0 | 0 | 1 |
| 208 | 0 | 0 | 1 | 1 | 1 | 0 | 1 | 0 | 1 | 0 | 1 | 0 | 0 | 1 | 0 | 1 | 0 | 0 | 1 | 1 | 1 | 1 | 1 | 1 | 1 |
| 209 | 0 | 0 | 1 | 0 | 1 | 0 | 1 | 0 | 0 | 0 | 1 | 0 | 0 | 1 | 0 | 1 | 0 | 0 | 1 | 1 | 0 | 0 | 1 | 1 | 1 |
| 210 | 0 | 0 | 1 | 0 | 0 | 0 | 0 | 0 | 1 | 1 | 0 | 0 | 0 | 1 | 0 | 0 | 0 | 0 | 1 | 0 | 0 | 0 | 1 | 0 | 1 |
| 211 | 0 | 1 | 1 | 0 | 0 | 1 | 0 | 0 | 0 | 0 | 1 | 1 | 0 | 1 | 1 | 1 | 0 | 0 | 1 | 0 | 1 | 1 | 0 | 1 | 0 |
| 210 | 0 | 0 | 1 | 0 | 0 | 0 | 0 | 0 | 1 | 1 | 0 | 0 | 0 | 1 | 0 | 0 | 0 | 0 | 1 | 0 | 0 | 0 | 1 | 0 | 1 |
| 211 | 0 | 1 | 1 | 0 | 0 | 1 | 0 | 0 | 0 | 0 | 1 | 1 | 0 | 1 | 1 | 1 | 0 | 0 | 1 | 0 | 1 | 1 | 0 | 1 | 0 |

**Key**

3a = Limited skilled human resources; 3b = Gaps in knowledge; 3c = Budgetary constraints; 3d = Lack of clear capacity development policy; 3e = Lack of formalized capacity-building programs; 3f = Limited study opportunities; 3g = Limited technical training opportunities; 3h = Insufficient coaching; 4a = Academic study opportunities; 4b = Seminars (physical/online; 4c = Physical training workshops; 4d = On-line training; 4e = International experts; 4f = Exposure visits; 4g = Coaching programs; 4h = Short courses; 4i = Review and planning; 5a = Hire a capacity-building consultant; 5b = Allocate a capacity-building budget; 5c = Create an individualized carrier development plan; 5d = Review the organization’s capacity-building policy; 5e = Incentives and rewards for capacity development achievements; 5f = Promote external partnerships and collaborations; 5g = Promote a learning culture; 5h = Implement regular training and development programs; 1 = yes; 0 = No.

#### **Appendix 5. Kaiser-Meyer-Olkin (KMO), and Bartlett's Test.**

|  | KMO | Bartlett's Test | | |
| --- | --- | --- | --- | --- |
|  |  | X^2^ | df | Sig. |
| Educational learning variables | 0.50 | 44.34 | 6 | p<0.001 |
| Organisational training variables | 0.503 | 36.53 | 6 | p<0.001 |
| Organisational coaching variable | 0.532 | 67.50 | 6 | p<0.001 |

KMO = Kaiser-Meyer-Olkin; X^2^ = Chi square; df = Degrees of freedom; sig = Significance

#### **Appendix 6. Demographic distribution of respondents and their association with gender.**

|  | **Gender N(%)** | | |
| --- | --- | --- | --- |
|  | **Male** | **Female** | **Total** |
| 1. **Age bracket** |  |  |  |
| less or equal to 30 | 10(66.7) | 5(33.3) | 15(10.6) |
| 31 to 40 | 26(54.2) | 22(45.8) | 48(34.0) |
| 41 to 50 | 26(46.4) | 30(53.6) | 56(39.7) |
| 51 or more | 13(59.1) | 9(40.9) | 22(15.6) |
| **Total** | **75**(**53.5**) | **66**(**46.5**) | **141(100)** |
| **X^2^ (P Value)** |  |  | **3.325^ns^** |
| 1. **Education level** |  |  |  |
| Diploma or less | 31(56.4) | 24(43.6) | 55(40.4) |
| Degree | 30(58.8) | 21(41.2) | 51(37.5) |
| Masters | 11(37.9) | 18(62.1) | 29(21.3) |
| Doctorate | 1(100.0) | 0(0.0) | 1(0.7) |
| **Total** | **73(53.7)** | **63(46.3)** | **136(100)** |
| **X^2^ (P Value)** |  |  | **4.487^ns^** |
| 1. **Management Level** |  |  |  |
| Admin | 10(83.3) | 2(16.7) | 12(9.0) |
| Research officer | 43(37.2) | 36(22.8) | 79(59.4) |
| Technician | 20(47.6) | 22(52.4) | 42(31.6) |
| **Total** | **73(53.5)** | **60(46.5)** | **133(100)** |
| **X^2^ (P Value)** |  |  | **9.476**** |
| 1. **Years in the organisation** |  |  |  |
| <5 | 18(75.0) | 6(25.0) | 24(17.1) |
| 5 to 10 | 9(81.8) | 2(18.2) | 11(7.9) |
| 10 to 20 | 38(43.2) | 50(56.8) | 88(62.9) |
| ≥21 | 10(58.8) | 7(41.2) | 17(12.1) |
| **Total** | **75(53.6)** | **65(46.4)** | **140(100)** |
| **X^2^ (P Value)** |  |  | **11.967***** |

*Source: Research Data* *Overall response rate for all respondents; N = Frequency, X^2^ = Chi-squared; ns = non-significant; *; **; *** = significant at 10%, 5% and 1% confidence levels.

#### **Appendix 7. Initial Eigenvalues of the variance explained by PCA.**

| Component | Educational learning | | | Organisational training | | | Organisational coaching | | |
| --- | --- | --- | --- | --- | --- | --- | --- | --- | --- |
|  | Total | % of Variance | Cumulative % | Total | % of Variance | Cumulative % | Total | % of Variance | Cumulative % |
| 1 | 1.476 | 36.890 | 36.890 | 1.481 | 37.033 | 37.033 | 1.779 | 44.476 | 44.476 |
| 2 | 1.284 | 32.109 | 69.000 | 1.095 | 27.370 | 64.403 | 0.977 | 24.427 | 68.904 |
| 3 | .681 | 17.025 | 86.024 | .896 | 22.394 | 86.797 | 0.814 | 20.344 | 89.248 |
| 4 | .559 | 13.976 | 100.000 | .528 | 13.203 | 100.000 | 0.430 | 10.752 | 100.000 |
